# The Relationship Between Maternal Exposure to Endocrine-Disrupting Chemicals and the Incidence of Congenital Heart Diseases: A Systematic Review and Meta-Analysis

**DOI:** 10.3390/metabo14120709

**Published:** 2024-12-16

**Authors:** Yasir Hassan Elhassan, Fahad Alahmadi, Emad Ali Albadawi, Abdullah Albarakati, Azizah Hendi Aljohany, Naweed SyedKhaleel Alzaman, Muayad Albadrani

**Affiliations:** 1 Department of Basic Medical Sciences, College of Medicine, Taibah University, Madinah 42353, Saudi Arabia; 2 Department of Women and Child Health, College of Medicine, Taibah University, Madinah 42353, Saudi Arabia; 3 Department of Medicine, College of Medicine, Taibah University, Madinah 42353, Saudi Arabia; 4 Department of Family and Community Medicine and Medical Education, College of Medicine, Taibah University, Madinah 42353, Saudi Arabia

**Keywords:** congenital heart diseases, endocrine-disrupting chemicals, maternal exposure, meta-analysis, systematic review

## Abstract

Background: Congenital heart diseases are among the most common birth defects, significantly impacting infant health. Recent evidence suggests that exposure to endocrine-disrupting chemicals may contribute to the incidence of congenital heart diseases. This study systematically reviews and analyzes the association between maternal endocrine-disrupting chemicals exposure and congenital heart diseases. Methodology: This systematic review and meta-analysis followed the Cochrane Handbook and PRISMA guidelines. We included studies assessing the link between maternal exposure to various endocrine-disrupting chemicals and the incidence of congenital heart diseases without restricting the study design or exposure assessment methods. Data were extracted from four databases, including PubMed, Scopus, Web of Science, and Cochrane Library, up to June 2024. Quality assessment of observational studies was conducted using the Newcastle–Ottawa Scale. Statistical analysis was performed using RevMan software version 5.3, presenting results as odds ratios with 95% confidence intervals. Results: Fifty-nine studies were included in the meta-analysis. The pooled analysis revealed a significant association between maternal endocrine-disrupting chemical exposure and the incidence of congenital heart diseases when measured using human samples (odds ratio = 1.63, 95% confidence interval [1.35–1.97], *p* < 0.00001). Notably, exposure to heavy metals, polycyclic aromatic hydrocarbons, and perfluoroalkyl compounds was strongly associated with congenital heart diseases. However, non-sample-based methods showed no significant overall correlation (odds ratio = 1.08, 95% confidence interval [0.93–1.26], *p* = 0.30), except for housing renovation compounds, which were linked to a higher incidence of congenital heart diseases. Conclusions: Maternal exposure to specific endocrine-disrupting chemicals, particularly heavy metals and polycyclic aromatic hydrocarbons, significantly increases the risk of congenital heart diseases. These findings underscore the need for preventive measures to reduce endocrine-disrupting chemicals exposure during pregnancy and further research to elucidate the underlying mechanisms.

## 1. Introduction

Congenital heart diseases (CHDs) are a major public health concern as they are one of the most widespread congenital anomalies known to affect around 0.8% of all newborns [1]. CHDs are still assumed to be strongly associated with the increased odds of birth defect death, illness, and infant and childhood healthcare costs [2]. The incidence of CHDs differs significantly by subtype with notable geographical distinctions, particularly regarding severe and very severe CHDs [3].

The heart’s morphogenesis is a complicated process that commences quite early during embryogenesis (around the third gestational week). It proceeds through stages of the primitive heart tube formation, looping, septation, and finally to the four-chamber heart with great vessels [4]. Disruptions at any stage in this process may lead to a wide variety of heterogeneous CHDs, ranging from simple defects like atrial septal defects to quite complex malformations like the Fallot tetralogy [5]. The timing of these disturbances and their nature itself mainly determine the severity and type of CHD that will occur. For example, cardiac looping defects at weeks 4–5 lead to the transposition of the great arteries, whereas septation abnormalities at weeks 5–8 cause ventricular septal defects [6]. Temporal and spatial information on cardiac development is critical to understanding the effects of environmental exposures, including EDCs, on the incidence and types of CHDs [7].

Despite ongoing uncertainty concerning the etiology of CHDs, emerging evidence proposes that an interaction between inherited factors and environmental exposures could play a substantial role in developing CHD [8]. Genetic abnormalities are believed to cause about 30% of CHD cases, leaving the cause of the remaining 70% undefined [9]. Multiple reports suggested a notable association between environmental factors, such as endocrine-disrupting chemicals (EDCs), and the development of CHDs [2,10,11]. Occupational exposure to environmental chemicals, particularly during the periconceptional phase, exerts negative effects on the reproductive system of both genders and can result in adverse health outcomes in the offspring [12]. Animal investigations have delineated that exposure to EDCs disrupts the embryonic cardiovascular development in zebrafish and rodents [13].

Since the twentieth century, considerable focus has been directed toward the influence of environmental factors on CHDs, largely driven by the rapid advancements in industry and the considerable emission of large volumes of pollutants. EDCs are identified as exogenous compounds that have the potential to disrupt the function of the endocrine system, resulting in substantial disruptions in hormonal functions such as synthesis and metabolic processes [14,15]. EDCs, which are otherwise known as endocrine-disrupting chemicals and pose a risk in most working environments, include some of the most common substances such as pesticides, phthalates, polycyclic aromatic hydrocarbons, bisphenol A, alkylphenols, and heavy metals. They hold these attributes because they are widely used as detergents, solvents, and other reagents in several manufacturing activities. Such compounds as EDCs can also be bought from some manufacturers in the form of widely used industrial products, including dyes, adhesives, paints, inks, dry cleaning solvents, and pharmaceutical preparations [2]. Emerging epidemiological evidence outlines a potential correlation between maternal EDC exposure and CHD [16,17]. In their meta-analysis, Dai et al. [10] reported a significant correlation between maternal EDC exposure and CHD development. Nevertheless, owing to the restrictions on their inclusion criteria, there are only a small number of studies (17 studies) that measured maternal exposure to EDCs in human biospecimens and most only investigated a few substances, mainly heavy metals. These studies do not address phthalates, polychlorinated compounds, and solvents. Additionally, they do not address the correlation between maternal exposure to EDCs measured using questionnaires, dietary assessments, and residential proximity to chemicals or drinking water and CHD development. Our study aims to address these limitations and provide comprehensive evidence concerning the correlation between maternal exposure to EDCs and the incidence of CHDs by including all available substances with no restrictions regarding specific measurement methods.

## 2. Methods

Our study strictly adhered to Cochrane Handbook rules [18]. This systematic review and meta-analysis was conducted in accordance with the PRISMA (Preferred Reporting Items for Systematic Reviews and Meta-Analyses) guidelines. The PRISMA checklist and flow diagram have been included in the Supplementary Table S1 [19].

### 2.1. Inclusion Criteria

We encompass all the relevant articles that assess the linkage between maternal exposure to EDCs and the incidence of CHD with no restriction regarding specific study designs or certain exposure assessment methods. The EDCs involved several substances such as pesticides, solvents, polychlorinated compounds, phthalates, alkyl phenolic compounds (APC), biphenolic compounds (BPC), heavy metals, and polycyclic aromatic hydrocarbons (PAHs). The outcome was CHD or its subtypes, like septal heart defects; including ventricular heart defect (VSD); atrial septal defect (ASD); conotruncal heart defects involving the tetralogy of Fallot (TOF); the transposition of great arteries (TGA); and obstructive heart defects, including left ventricular outflow tract obstruction defects (LVOTODs), right ventricular outflow obstruction defects (RVOTODs), and other heart defects like patent ductus arteriosus (PDA) and total anomaly pulmonary venous return (TAPVR).

Additionally, we only included studies that reported adjusted odds ratio (a.O.R.) or crude odds ratio (c.O.R) or provided data to calculate them. If the study reported a.O.R and c.O.R, we only extracted the a.O.R to decrease the effect of confounding variables and more accurately represent the correlation between maternal EDC exposure and the incidence of CHD.

We excluded review articles, conference abstracts, single-arm studies, and foreign language studies.

### 2.2. Literature Search and Selection Process

Four databases, namely PubMed, Scopus, Web of Science, and Cochrane Library, were systemically searched from study inception until June 2024. Our search strategy was a mixture of keywords related to EDCs, like pesticides, solvents, phthalates, and heavy metals, and to CHDs, like VSD, ASD, LVOTODs, RVOTODs, tetralogy of Fallot, and PDA. Supplementary Table S2 contains the comprehensive search strategy for each database.

The screening was conducted in two phases. First, the title and abstracts of obtained reports were evaluated using a Rayyan web [20]. Then, we retrieved the full text of eligible studies and evaluated them based on our specific criteria. Duplicates were eliminated utilizing Endnote (Clarivate Analytics, Philadelphia, PA, USA).

### 2.3. Data Collection

Utilizing a pre-designed extraction sheet, we extracted the following items: study design, country, data sources, sample size, method of exposure assessment, main chemicals, and conclusion.

### 2.4. Quality Evaluation

We assessed the quality of observational studies utilizing the Newcastle–Ottawa (NOS) scale [21]. The NOS assesses the risk of bias concerning three main items: selection of the cohorts, comparability between the cases and controls, and exposure assessment. NOS classify the studies as having good, moderate, or poor quality according to the cumulative score.

### 2.5. Statistical Analysis

The relationship between EDCs and the incidence of CHDs was presented as O.R. with its corresponding confidence interval (C.I.). We utilized a random-effect meta-analysis model during the pooling of effect sizes. A significant outcome was identified when *p*-value < 0.05. A chi-square-*p* value of <0.1 or an I-square of more than 50% was considered as significant heterogeneity. Additionally, we grouped the analysis based on the type of EDCs to pesticides, phthalates, PAHs, heavy metals, and other compounds not grouped under any of the mentioned substances. We also classified the analysis according to the method of the exposure assessment as sample-based exposure assessment, involving blood sample, hair sample, urine sample, umbilical cord sample, fetal placental tissue sample, and non-sample-based exposure assessment, including questionnaire-based assessment, dietary assessment based on FFQ items, and residential proximity to chemicals or drinking water. Publication bias was assessed utilizing a funnel plot. All the analyses were executed using Windows RevMan software (version 5.3).

## 3. Results

### 3.1. Literature Search

**Figure 1:**
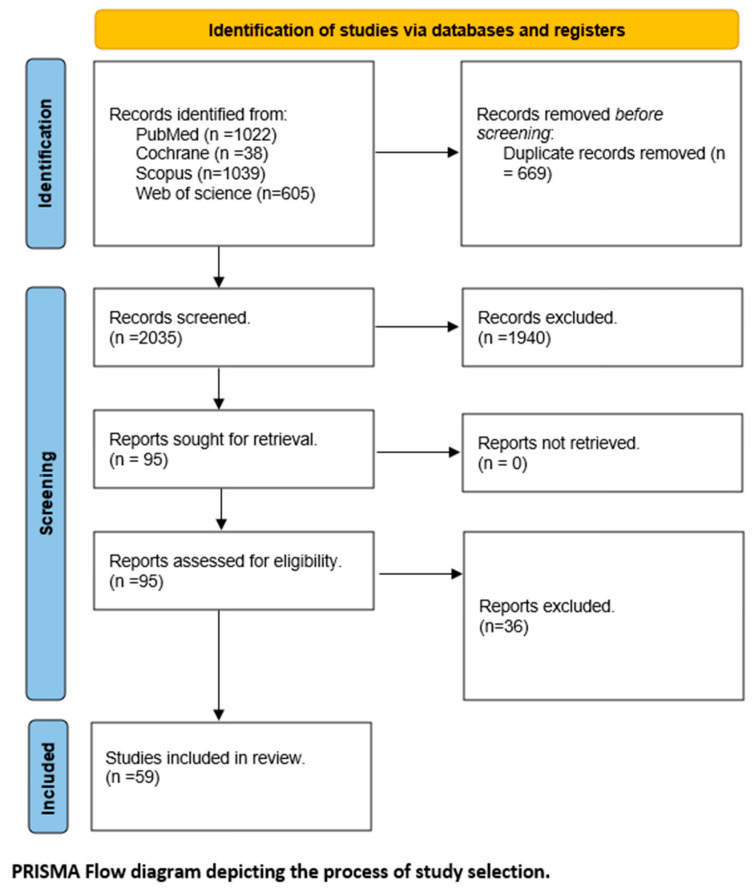
PRISMA Flow Chart: This figure illustrates the flow of information through the different phases of the systematic review. It includes the number of records identified, screened, eligible, and included in the meta-analysis, along with reasons for exclusions at each stage.

Our primary electronic search of the four databases yielded 2704 articles; of these, 669 articles were eliminated as duplicates using Endnote. Thus, 2035 records were available for the title and abstract screening phase. Then, 1940 articles were eliminated after the title and abstract screening. Finally, 95 reports were included in the full-text screening phase, of which 59 studies were involved in our meta-analysis. The PRISMA flow diagram of the review selection phase is presented at Figure 1.

### 3.2. Summary of the Included Studies

Fifty-nine articles were ultimately included in our investigation [2,11,16,22,23,24,25,26,27,28,29,30,31,32,33,34,35,36,37,38,39,40,41,42,43,44,45,46,47,48,49,50,51,52,53,54,55,56,57,58,59,60,61,62,63,64,65,66,67,68,69,70,71,72,73,74,75,76,77]. The study designs of our included studies were observational, with fifty-four case–control studies and five cohort studies. The included studies were carried out in several countries, mainly China and the USA, with a total of 29 and 15 studies, respectively. The methods of exposure assessment varied across the included studies, such as human samples, including blood, hair, and urine samples, and other methods, including questionnaires, residential proximity to the chemicals, and dietary assessment based on FFQ items. A comprehensive summarization of the characteristics of the retrieved reports is outlined in Table 1.

### 3.3. Quality Assessment of the Included Studies

The quality of case–control studies varied from good to moderate. The majority of studies showed a good definition of the case and a good representation of the community. The detailed quality evaluation for case–control studies is shown in Supplementary Table S3.

Five cohort studies were involved in our study: three were of good quality and two were of moderate quality. The quality assessment of cohort studies is thoroughly detailed in Supplementary Table S4.

### 3.4. Study Outcomes

#### 3.4.1. Any Congenital Heart Defects

**Figure 2:**
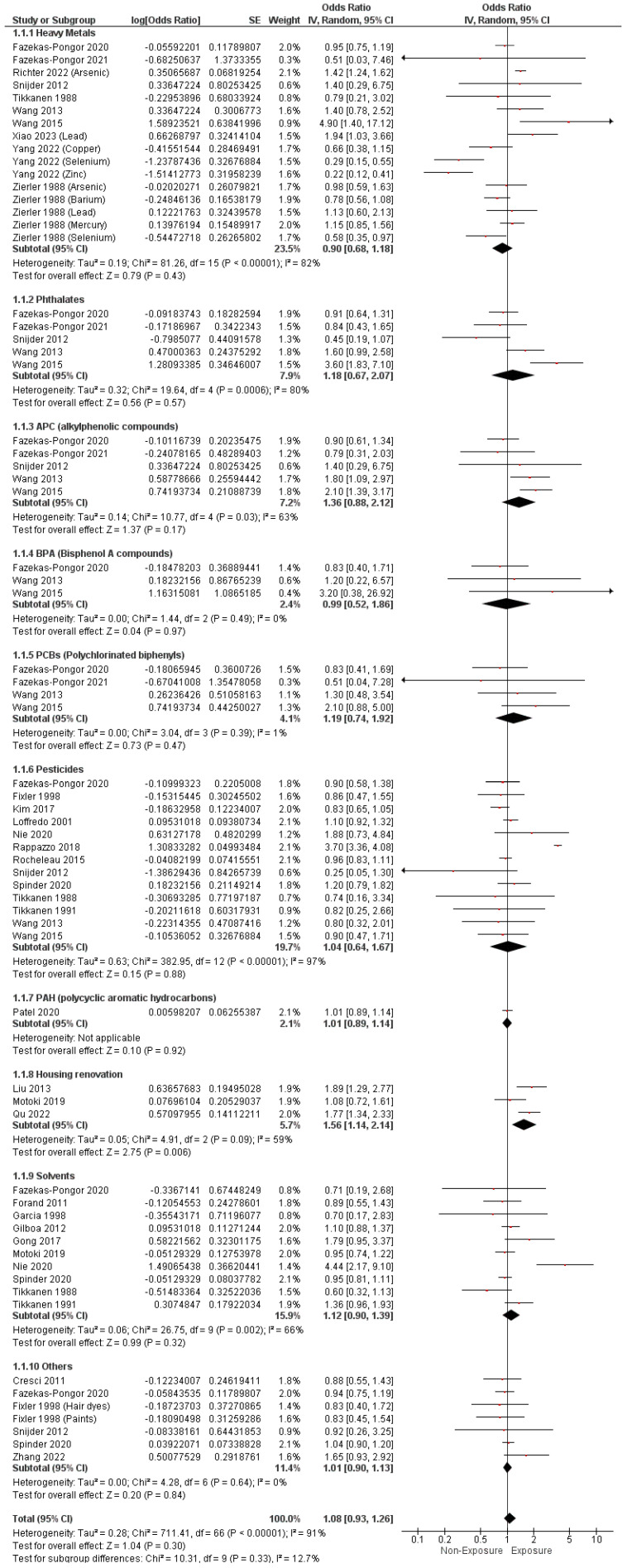
Forest plot of congenital heart defect incidence measured by non-sample methods. This forest plot shows the odds ratios and 95% confidence intervals for the association between maternal exposure to endocrine-disrupting chemicals and the incidence of congenital heart defects, based on non-sample methods of exposure assessment. Subgroups include heavy metals, phthalates, alkylphenolic compounds, bisphenol A, polychlorinated biphenyls, pesticides, polycyclic aromatic hydrocarbons, solvents, and housing renovation compounds.

Regarding EDCs measured by non-sample methods, the pooled analysis did not reveal any substantial correlation between maternal exposure to EDCs and the development of CHDs (O.R. = 1.08, 95% C.I. [0.93–1.26], *p* = 0.30). A considerable heterogeneity was noticed between the pooled results (I^2^ = 91%, *p* < 0.0001), as pointed out in Figure 2.

**Table 1:** Summary and baseline characteristics of the included studies.

Study ID	Country	Study Design	Data Sources	Recruitment Time	Number of CHD Cases	Number of Controls	Exposure Assessment Method	Chemicals	Definition of CHD Cases	Conclusion
Batra 2007 [22]	USA	Case–control	Washington hospitals	1987–2003	3489	13,290	Agricultural employment	Pesticides	“Cases were defined as liveborn singleton infants born between 1987 and 2003 who were diagnosed with VSD within the first 2 years of life. We identified these cases from the Comprehensive Hospital Abstract Reporting System using the International Classification of Diseases, Ninth Revision (ICD-9) code for VSD”.	“Although these findings suggest regional variation in Washington State in the occurrence of VSD, the basis for this variation remains to be determined”.
Brender 2014 [23]	USA	Case–control	Texas Birth Defects Registry	1996–2008	60,154	280,764	Residential proximity to chemicals	Solvents	“Congenital heart defects were identified from the Texas Birth Defects Registry (TBDR) for births occurring during 1996–2008”.	“These findings suggest that maternal residential proximity to industrial emissions of chlorinated solvents might be associated with selected birth defects in offspring, especially among older mothers”.
Carmichael 2014 [24]	USA	Case–control	The California Center of the National Birth Defects Prevention Study (NBDPS)	1997–2006	569	785	Residential proximity to chemicals	Pesticides	“Cases included infants or fetuses with CHDs confirmed by echocardiography, cardiac catheterization, surgery, or autopsy reports”.	“Most pesticides were not associated with increased risk of specific heart defect phenotypes. For the few that were associated, results should be interpreted with caution until replicated in other study populations”.
Cresci 2011 [25]	Italy	Case–control	Pediatric cardiac centers	2008–2010	180	180	Questionnaire	EDCs	“Isolated CHDs”	“Paternal smoking and exposure to toxicants for both parents affect the risk of children with CHD. Polymorphisms in GST genes can modify a person’s risk of toxicant exposure-induced disease”.
Fazekas-Pongor 2021 [27]	Hungary	Case–control	Hungarian Case–Control Surveillance of Congenital Abnormalities Study	1997–2002	2263	6789	Job–exposure matrix	Pesticides, polychlorinated organic compounds, phthalates, alkyl phenolic compounds, heavy metals, biphenolic compounds	“Cases were liveborn infants affected by any CHD”.	“We identified that certain occupations may increase the occurrence of certain congenital heart disease phenotypes in the offspring. By paying closer attention to those working in these areas, antenatal detection rates of congenital heart diseases may be improved”.
Fazekas-Pongor 2021 [26]	Hungary	Case–control	Hungarian Case–Control Surveillance of Congenital Abnormalities Study	2007–2008	577	1731	Job–exposure matrix	Pesticides, polychlorinated organic compounds, phthalates alkylphenolic compounds, heavy metals	“Only live births reported to the HCAR within the first 3 months of CHD diagnosis are recruited”.	“Even though parental occupational exposure to EDCs seems to have a minor impact on the occurrence of CHDs, the results of biological and environmental monitoring should be taken into consideration as well”.
Fixler 1998 [28]	USA	Case–control	Six major newborn nurseries in Dallas, the two cytogenetic laboratories in the area, direct referrals from medical practitioners, the Down Syndrome Guild (a parent support group) and the genetics and pediatric cardiology services at Southwestern Medical Center	-	89	82	Questionnaire	Paints, hairdyes, pesticides	“A case was defined as an infant who had cytogenetic confirmation of trisomy 21 (primary nondisjunction) and associated congenital heart disease confirmed by pediatric cardiology consultation”.	“In summary, the present study found relatively small differences in prenatal exposures between cases and controls and low exposure rates for many agents. The results show that only a limited number of substances had differences in exposure rates between case and control mothers as high as 5% or more”.
Forand 2011 [29]	USA	Cohort	New York State Vital Statistics files (NYSDOH) Congenital Malformations Registry (CMR)	1978–2002	20	-	Residential proximity to chemicals	Solvents	“All cardiac defects identified using these codes ICD-9 codes 745.0–747.9”	“Maternal residence in both areas was associated with cardiac defects. Residence in the TCE area, but not the PCE area, was associated with LBW and fetal growth restriction”.
Garcia 1998 [30]	Spain	Case–control	Eight public hospitals in Spain	1993–1994	60	255	Questionnaire	Solvents	“All cardiovascular defects identified using these codes ICD-9 codes 745.0–747.9”	“These data are compatible with an increased risk for oral clefts in the offspring of women working in the leather industry. Some other categories of defect could have an increased risk as well, although our data cannot exclude random error as an explanation. Given these results and previous Findings in similar studies, some precautionary recommendations regarding maternal exposure in leather industries, probably in relation to solvents, would be justified”.
Gilboa 2012 [31]	USA	Case–control	National Birth Defects Prevention Study (NBDPS)	1997–2002	2047	2951	Job–exposure matrix	Solvents	“All CHD cases were confirmed by echocardiography, cardiac catheterization, surgery, or autopsy”.	“The authors found evidence of associations between occupational exposure to solvents and several types of CHDs. These results should be interpreted in light of the potential for misclassification of exposure”.
Gong 2017 [32]	China	Cohort	Guangzhou Panyu Maternity Hospital	-	145	5236	-	Solvents	-	“Hazardous substances in factories, especially organic solvents, were identified as potential risk factors for CHDs. Besides, exposure to high noise also increased the incidence of CHDs”.
Hu 2014 [34]	China	Case–control	Four tertiary hospitals in China	2010–2011	212	212	Hair sample	Heavy metals (zinc), heavy metals (copper)	“Fetuses had been diagnosed with a CHD by echocardiography were recruited in the case group”.	“Women with excessive copper concentrations have a significantly increased risk of having offspring with a CHD. A low maternal zinc status might have a correlation with CHDs, and an interaction between copper and zinc might exists, but an epidemiological study with a larger sample size is needed to confirm this finding”.
Huang 2023 [11]	China	Case–control	Birth cohort study conducted at the Gansu Provincial Maternity and Child Care Hospital during 2010–2012 in Lanzhou, China.	2010–2012	97	194	Blood sample	Heavy metals (lead)	“The cases were newborns with CHDs and his mother”.	“Overall, our study suggests a significant association between pregnancy Pb exposure and risk of CHDs, especially for isolated CHDs. Future studies are needed to elucidate the underlying mechanism”.
Jin 2016 [33]	China	Case–control	“Four different maternal and children’s hospitals in China”.	2010–2011	339	333	Hair sample	Heavy metals (arsenic), heavy metals (cadmium)	“A case was identified as a pregnant woman whose fetus was diagnosed with a congenital heart defect (CHD) via echocardiography, with the CHD diagnosis confirmed after delivery”.	“Our research indicates that exposure to arsenic and cadmium during pregnancy could significantly increase the risk of congenital heart defects (CHDs) in offspring. Cadmium appears to exacerbate the relationship between arsenic exposure and CHD risk in offspring”.
Kim 2017 [35]	USA	Case–control	Texas Birth Defects Registry	1999–2005	18,291	4414	Residential maternal address to corresponding water supply	Pesticides (atrazine)	“In this study, cases were defined as subjects with any structural CHD (BPA codes: 745.000–745.490, 745.510–745.900, 746.000–746.995, 747.100–747.320, 747.330–747.490)”.	“These findings should be interpreted with caution in light of potential misclassification and relatively large proportions of subjects with missing atrazine data. Thus, more consistent and complete monitoring and reporting of drinking water contaminants will aid in better understanding the relationships between pesticide water contaminants and birth defects”.
Li 2018 [36]	China	Case–control	“Six tertiary maternal and child health hospitals”	2010–2015	357	270	Urine sample	Polycyclic aromatic hydrocarbons	“CHDs were confirmed in the fetuses by appointed senior ultrasonic doctors. For the CHDs-affected fetuses that were aborted, CHDs were further confirmed by humanitarian examination of the pathological anatomy. For the CHDs-affected fetuses that were born, a further ultrasound examination was performed within 30 postnatal days”.	“Our study suggests that maternal AHR polymorphisms may modify the association of PAHs exposure with CHDs, CYP1A2 or CYP2E1 polymorphisms significantly interact with PAHs exposure on CHDs”.
Li 2024 [37]	China	Case–control	“Birth cohort study was conducted (2010–2012) at the Gansu Provincial Maternity and Child Care Hospital in Lanzhou, northwest of China”.	2010–2012	97	194	Blood sample—Umbilical cord blood	Heavy metals (aluminum), heavy metals (iron)	“The cases were newborns with CHDs and his mother”.	“Our study suggests that exposure to Al during pregnancy (≥2408 mg/L) is significantly associated with an increased risk of CHDs in offspring, especially septal defects and that high levels of Al and Fe are strongly correlated with fetal heart development. Further research is needed to understand the underlying mechanisms”.
Liu 2013 [38]	China	Case–control	“Four tertiary maternal and child hospitals (Guangdong, Fujian, Henan and Hubei provinces) in China”	2010–2011	346	408	Questionnaire	-	“CHD was defined as “a gross structural abnormality of the heart or intrathoracic great vessels that is actually or possibly of functional significance”, as described by Mitchell”.	“Maternal exposure to housing renovations may have an increased risk of giving birth to fetuses with some selected types of CHD. This relationship was stronger for women who moved into a newly decorated house. However, considering the limited number of subjects and the problem of multiple exposures, more research is needed to clarify the effects seen here”.
Liu 2015 a [40]	China	Case–control	“Four maternal and child tertiary hospitals in China”	2010–2011	223	223	Hair sample	Heavy metals (aluminum)	“Eligible fetuses with cardiac defects diagnosed during prenatal examination were recruited as the case group”.	“A high maternal aluminum concentration may significantly increase the risk of delivering a child with a CHD, such as a septal defect, conotruncal heart defect and right-side obstruction”.
Liu 2015 b [39]	China	Case–control	“Four maternal and child hospitals in China”	2010–2011	316	348	Hair sample	Heavy metals (lead)	“Fetuses diagnosed prenatally with cardiac defects were recruited as the case group”.	“Maternal exposure to lead is linked to an increased risk of certain subtypes of congenital heart defects (CHDs) in offspring. Additionally, there is evidence suggesting a potential dose–response relationship”.
Loffredo 2001 [41]		Case–control	The Baltimore-Washington Infant Study	1987–1989	1001	771	Questionnaire	Pesticides	“Cases were defined as infants born alive in 1981–1989 to parents who were residents of the study area. All six pediatric cardiology centers in the region Participated in the study. The diagnosis of specific structural heart disease was confirmed before 1 year of age by pediatric cardiologists”.	“These results raise new questions about the possible epidemiologic association of TGA with some classes of pesticides and warrant new, carefully targeted investigations”.
Luan 2023 [42]	China	Case–control	The specialized cardiovascular center is in Guangdong Province, South China.	2014–2017	41	74	Blood sample	Pesticides (triclosan)	“Fetuses in the case group were confirmed to have major congenital heart defects after birth through echocardiographic examination, following the International Classification of Diseases criteria”.	“Our findings indicated that at normal exposure levels, the increase of maternal blood TCS concentration may have an inverse association with CHD, which merits further investigation”.
Lupo 2012 [43]	USA	Case–control	National Birth Defects Prevention Study (NBDPS)	1997–2002	1907	2853	Questionnaire	Polycyclic aromatic hydrocarbons	“All CHD cases were confirmed by echocardiography, cardiac catheterization, surgery or autopsy”.	“Our findings do not support an association between potential maternal occupational exposure to PAHs and various CHDs in a large, population-based study. For CHD phenotypic subtypes in which modest nonsignificant associations were observed, future investigations could be improved by studying populations with a higher prevalence of PAH exposure and by incorporating information on maternal and fetal genotypes related to PAH metabolism”.
Motoki 2019 [44]	Japan	Cohort	Japan Environment and Children’s Study	2011–2014	756	66,113	Questionnaire	Solvents	“CHD diagnosed by obstetricians up to 1 month after birth”.	“This large nationwide survey provides important information on a possible association of house renovation during pregnancy with congenital male genital abnormality which needs confirmation in future studies”.
Nana Li 2024 [45]	China	Case–control	“Six tertiary maternal and child health hospitals with the qualification of performing prenatal diagnoses located in Fuzhou, Nanning, Chengdu, Zhengzhou, Wuhan, and Shenzhen city”.	2010–2015	245	268	Urine sample	Phthalates	“The cases group were pregnant women whose fetuses were diagnosed with CHDs by echocardiography, and no any extracardiac abnormalities and gestational age more than 12 weeks”.	“The polymorphisms of maternal UGT1A7 gene at rs4124874 and rs887829 were significantly associated with an increased risk of CHDs. More large-scale studies or prospective study designs are needed to confirm or refute our findings in the future”.
Nie 2020 [46]	China	Case–control	The Guangdong Registry of Congenital Heart Disease (GRCHD)	2004–2014	4726	4726	Questionnaire	Pesticides, solvents	“CHDs were defined and coded according to the International Classification of Diseases (ICD-10 CA codes Q20.000–Q28.000). A team of obstetricians, pediatricians, or pediatric cardiologists examined all newborns before discharge or within 72 h after birth. Two specialty-trained cardiologists reviewed all echocardiograms of CHDs cases”.	“Paternal alcohol consumption and multiple paternal factors were significantly associated with CHDs in China. Paternal smoking and low SES factors modified paternal alcohol consumption–CHDs relationships. Further studies are needed to confirm these findings”.
Nie 2024 [47]	China	Case–control	Guangdong People’s Provincial Hospitals	2014–2018	164	164	Blood sample	Heavy metals	“All newborns diagnosed with CHD in utero received postnatal B-mode echocardiography to verify the diagnosis. Live births without prenatal detection of CHD were systematically assessed prior to discharge to ascertain the need for additional evaluation for probable CHD”.	“This study is the first to report an association between elevated levels of mixed trace elements in maternal plasma with an increased prevalence of fetal CHDs, particularly in the case of Pb and Sn. Findings from this study provide further evidence of the important of heavy metal pollution to human health. They can help stakeholders prioritize policies and develop interventions to target the leading contributors to human exposure”.
Ou 2017 [48]	China	Case–control	Provincial General Hospital in China	2012–2013	112	107	Blood sample	Heavy metals	“Patients were diagnosed with CHDs after review by two physicians specialty-trained in echocardiography”.	“Our results suggest that Pb exposure poses an important health threat even under the current standard for protecting human health (10 μg/dL). These data can be used for developing interventions and identifying high-risk pregnancies”.
Ou 2021 [49]	China	Case–control	Single Provincial Hospital in Guangzhou, China	2014–2018	158	158	Blood sample	Perfluoroalkyl	“At least two senior pediatric cardiologists confirmed each CHD diagnosis after delivery, using B-mode echocardiography, computed tomography, cardiac catheterization, or surgery, as clinically necessary. CHD cases were coded as Q20–Q28 based on the International Classification of Diseases version 10 (ICD-10)”.	“These exploratory findings suggested that gestational exposure to most PFAS, especially linear PFOS, 6 m-PFOS, PFDA, and PFDoA, was associated with greater risks for septal and conotruncal defects. However, a larger, adequately powered study is needed to confirm our findings and to more comprehensively investigate the potential teratogenic effects of other more recently introduced PFAS, and on associations with individual CHD subtypes”.
Patel 2020 [50]	USA	Case–control	National Birth Defects Prevention Study (NBDPS)	1997–2011	4775	7734	Questionnaire	Polycyclic aromatic hydrocarbons	“All CHD cases were confirmed by echocardiography, cardiac catheterization, surgery, or autopsy”.	“Women in the highest quartile of estimated cumulative occupational PAH exposure during early pregnancy were more likely to have offspring with conotruncal heart defects, specifically ToF, compared to women with no occupational PAH exposure. Other comparisons between PAHs and other CHDs subgroups did not show any statistically precise associations”.
Qu 2023 [51]	China	Case–control	Six tertiary A hospitals in Xi’an, Shaanx	2014–2016	587	1180	Questionnaire	-	“Singleton pregnancy, End of pregnancy was between January 2014 and December 2016, Mothers whose fetuses or children were diagnosed with isolated CHDs from 28 weeks of pregnancy to 28 days after birth”.	“Our study suggests that maternal exposure to housing renovation during the periconceptional period was associated with an increased risk of isolated CHD in offspring. Consequently, it would be beneficial to avoid living in a renovated home from 12 months before pregnancy through the first trimester to lower isolated CHD in infants”.
Qu 2024 [52]	China	Case–control	Birth cohort study in China	2014–2017	141	282	Blood sample	Pesticides	“All participants in the parent birth cohort underwent active screening for fetal cardiac anomalies using basic ultrasound at 18–24 gestational weeks as part of routine prenatal care. Fetuses suspected of having CHDs were referred for echocardiography at 22–26 gestational weeks to confirm the diagnosis”.	“Pregnant women were primarily exposed to N-dm-ACE and IMI during early-mid pregnancy. Gestational exposure to NEOs may be associated with an increased risk of septal defects, but the evidence is limited at present. Education is a potential contributing factor to NEO exposure in pregnant women. Larger and more precise studies with longitudinal biospecimen collection are recommended to validate our exploratory findings”.
Rappazzo 2018 [53]	USA	Case–control	North Carolina Birth Defects Monitoring Program	2003–2005	2459	298,548	Residential proximity to chemicals	Pesticides	“Cases were liveborn singleton infants diagnosed by cardiovascular defects”.	“Our results suggest differing patterns of association for birth defects with residential exposure to seven pesticide-active ingredients in North Carolina”.
Richter 2022 [54]	Denmark	Cohort	Danish Medical Birth Register	1997–2014	10,627	-	Maternal residential address proximity to corresponding water supply	Heavy metals	“Information on congenital malformations identified within the first year of life including CHD was obtained from The Danish Medical Birth Register. Diagnoses in the Danish Medical Birth Register have since 1997 been recorded according to the International Classification of Disease (ICD), version 10 (ICD-10 codes)”.	“The findings indicate that maternal exposure to arsenic in drinking water, even at low concentrations (i.e., 0.5–0.9 μg/L) increased the risk of congenital heart disease in the offspring”.
Rocheleau 2015 [16]	USA	Case–control	National Birth Defects Prevention Study (NBDPS)	1997–2000	3318	2979	Job–exposure matrix	Pesticides	“CHD cases were selected through medical record review, excluding syndromes and chromosomal abnormalities. Pediatric cardiologists classified CHDs by phenotype and complexity, distinguishing simple, associated, and complex defects. Cases were further categorized based on the presence or absence of major extracardiac defects, ensuring a comprehensive understanding of CHD diversity”.	“Broad pesticide exposure categories were not associated with CHDs overall, but examining specific CHD subtypes revealed some increased odds ratios. These results highlight the importance of examining specific CHDs separately. Because of multiple comparisons, additional work is needed to verify these associations”.
Rudnai 2014 [55]	Hungary	Case–control	Hungarian Congenital Anomalies Registry	1987–2003	3204	5880	Maternal residental address proximity to corresponding water supply	Heavy metals	“Congenital anomalies of the circulatory system (International Classification of Diseases, ICD-10 codes: Q20.0–Q28.9) were considered as cases”	“The findings presented indicate a heightened risk of congenital heart anomalies in infants whose mothers were exposed to drinking water containing arsenic levels exceeding 10 g/L during pregnancy. Further investigation into potential analogous effects of concentrations below 10 g/L is recommended”.
Sainan Li 2024 [56]	China	Case–control	Environmental multicenter case–cohort study in China.	2016–2021	185	247	Blood sample	Perfluoroalkyl	“A pregnant woman whose fetus was diagnosed with CHD was enrolled as a case”.	“These findings provide essential evidence that there is indeed a health crisis associated with PFASs and that it is linked to CHD”.
Salehi 2022 [57]	Iran	Case–control	Pediatric clinic of Vali-e-Asr Hospital in Birjand, Iran	-	100	146	Blood sample	Heavy metals	“The case group was ascertained through echocardiography and angiography screening tests of babies between birth date and 6 months”.	“The results of our study showed that mothers of children with CHD had higher blood concentrations than mothers of healthy children”.
Shaw 1999 [58]	USA	Case–control	California birth defects monitoring program	1997–1988	207	734	Job–exposure matrix	Pesticides	“Live born infants and fetal deaths diagnosed by conotruncal heart defects”.	“Despite investigating multiple sources of potential pesticide exposure, we could not adequately discriminate whether the observed effects are valid or whether biased exposure reports elevated risks of congenital anomalies”.
Snijder 2012 [59]	The Netherlands	Case–control	The HAVEN study	2003–2010	424	480	Job–exposure matrix	Pesticides, phthalates, alkylphenolic compounds, heavy metals	“Children diagnosed with CHD within the first 15 months after birth by pediatric cardiologists were identified from the hospital registry and invited to participate”.	“Periconceptional paternal (but not maternal) occupational exposure to certain chemicals is associated with an increased risk of CHDs in children. The results, however, must be interpreted cautiously as exposure probabilities are a crude measure of exposure”.
Spinder 2020 [60]	The Netherlands	Case–control	European Registration of Congenital Anomalies and Twins Northern Netherlands (EUROCAT NNL)	1997–2013	1174	5602	Job–exposure matrix	Solvents, pesticides	“CHD cases were coded by trained registry staff according to the International Classification of Diseases 9th revision (ICD-9) until 2001 and according to ICD 10th revision (ICD-10) from 2002 onwards, using international EUROCAT guidelines”.	“Women should take preventive measures or avoid exposure to mineral and organic dust as well as metal dust and fumes early in pregnancy as this could possibly affect fetal heart development”.
Suhl 2022 [61]	USA	Case–control	National Birth Defects Prevention Study (NBDPS)	1997–2011	6483	10,446	Dietary assessment based on the FFQ items	Heavy metals	“Clinical geneticists classified case children with a CHD by phenotype level, defect complexity, and extracardiac defects”.	“Exploration of maternal dietary exposure to total and inorganic arsenic and CHDs produced few positive associations but was limited by available food item concentrations. Future research requires expanded collection of dietary data, improved estimates of concentrations, and consideration of non-dietary sources of arsenic exposure”.
Sun 2022 [62]	China	Case–control	Birth cohort study in t the Gansu Provincial Maternity and Child Care Hospital China.	2010–2012	97	194	Blood sample, umbilical cord blood	Heavy metals	“Children diagnosed with CHDs”.	“Titanium can pass through the placental barrier, and the occurrence of CHDs may be associated with exposure to titanium”.
Tao 2019 [63]	China	Case–control	Sex tertiary maternal and Child Hospitals in China	2010–2015	357	270	Urine sample	Polycyclic aromatic hydrocarbons	“Cases were restricted to pregnant women having fetuses diagnosed with CHDs and without any extracardiac abnormalities determined by echocardiography and having a gestational age greater than 12 weeks”.	“A potential modifying effect of PAH exposure on genetic polymorphisms of EPHX1 was observed in susceptibility to CHDs. However, no multiplicative-scale interactions were detected between maternal PAH exposure and EPHX1 gene polymorphisms in influencing CHD risk. Further assessment of the role of EPHX1 gene polymorphisms in CHDs is warranted, particularly in interaction with PAH exposure”.
Tikkanen 1988 [64]	Finland	Case–control	Finnish Register of Congenital Malformations	1980–1981	202	158	Questionnaire	Heavy metals, solvents, pesticides	“The diagnoses of the liveborn children were evaluated by a pediatric cardiologist”.	“Comparing mothers who used medications in the first trimester with those who did not showed an odds ratio of 2.2 (95% confidence interval (1.3–3.9) when adjusted for potential confounding by multivariate logistic methods”.
Tikkanen 1991 [65]	Finland	Case–control	Finnish Register of Congenital Malformations	1982–1984	573	1055	Questionnaire	Pesticides, solvents	“The cardiovascular anomaly detected during the first year of life was diagnosed by echocardiographic examination, cardiac catheterization, surgery, or autopsy”.	“It is concluded that some common environmental exposures during early pregnancy to physical and chemical factors should not necessarily be considered hazardous for the developing fetal heart. The causes of the majority of cardiovascular malformations remain unknown”.
Tikkanen 1992 [66]	Finland	Case–control	Finnish Register of Congenital Malformations	1982–1983	406	756	Questionnaire	Solvents	“The cases were taken from all infants diagnosed with cardiovascular malformations”.	“It is concluded that many maternal exposures at work seem not to have a teratogenic effect on the fetal heart, although the limited power of this investigation needs to be borne in mind”.
Tikkanen 1993 [67]	Finland	Case–control	Finnish Register of Congenital Malformations	1982–1983	50	756	Questionnaire	Solvents	“The CoA material was derived from all infants born in Finland during 1982–1983 (132,993 births) who had a cardiovascular malformation diagnosed between the 20th week of gestation and 1 year of age”.	“The risk of CoA was not associated with seasonal variation, maternal smoking, alcohol consumption, or use of deodorants. It is concluded that genetic factors explain only a small fraction of the causes of CoA and that many common environmental exposures during early pregnancy are unlikely to be real risk factors for CoA. However, the power of this study was weak for testing the teratogenicity of specific chemicals”.
Villasenor 1991 [68]	USA	Case–control	The Baltimore-Washington Infant Study	1981–1987	41	2801	Questionnaire	Heavy metals, solvents, pesticides	“Among the resident live births of a defined study area (Maryland, the District of Columbia, and Northern Virginia), all infants are ascertained in whom the cardiovascular disease has been confirmed before 1 year of age by echocardiography, cardiac catheterization, surgery, or autopsy”.	“Multivariate analysis suggested an interaction between pesticide exposure and family history and, thus, a possible familial susceptibility to environmental teratogens. Although the number of TAPVR cases is small, this epidemiologic study identifies hypotheses that may be further explored in morphogenetic and epidemiology studies”.
Wang 2013 [69]	China	Case–control	Department of Pediatric Cardiology in West China Second University Hospital	-	216	216	Job–exposure matrix	Pesticides, phthalates, polychlorinated organic compounds, alkylphenolic compounds, biphenolic compounds, heavy metals	“The diagnoses of all CHDs cases were confirmed by cardiac catheterization”.	“The C3435T polymorphism in the ABCB1 gene of the fetus elevates the risk of CHDs in a Han Chinese population, especially when mothers are exposed to phthalates and alkylphenolic compounds during the periconceptional period. This increased risk is particularly notable for septal defects”.
Wang 2015 [2]	China	Case–control	Two university medical centers: the West China Second University Hospital, Sichuan University in Chengdu, and the Luzhou Medical College Hospital in Luzhou	2012–2013	707	593	Job–exposure matrix	Pesticides, phthalates, polychlorinated organic compounds, alkylphenolic compounds, biphenolic compounds, heavy metals	“The diagnoses of all CHDs cases were confirmed by cardiac catheterization”.	“In conclusion, parental occupational exposures to some specific EDs, in particular phthalates and alkylphenolic compounds, are associated with an increased risk of some CHD phenotypes. However, the findings need to be considered more circumspectly regarding a crude measure of exposure probabilities and small numbers”.
Wang 2022 [70]	China	Case–control	Multicenter case–control study being carried out in 7 cities and one country of 8 provinces of China	-	303	303	Blood sample	Heavy metals	“The diagnosis of CHD was made by qualified sonographers with fetal echocardiography and coded according to the International Classification of Diseases version 10 (ICD-10)”.	“Using metal concentrations in maternal plasma obtained during the second or third trimester as exposure markers, we found that the risk of CHDs increased with the levels of the mixtures of As, Cd, Hg, Pb, and Mn, with Hg being the most important contributor to the mixture effect”.
Xiao 2023 [71]	China	Case–control	126 community health service centers in China	2019–2021	675	1545	Questionnaire	-	“All CHD cases were diagnosed from 28 weeks after pregnancy to 7 days by pediatric cardiologists after clinical diagnosis was performed by heart auscultation and fetal and neonatal echocardiography according to ICD-10 classification criteria”.	“Living near main roads and housing renovation during the periconceptional period are significantly associated with the increased risks for all CHDs and isolated CHDs. Further study is needed to extend sample size to explore the effects of time and frequency of burning incense and family relationships on CHDs in offspring”.
Yang 2022 [72]	China	Case–control	Six tertiary comprehensive hospitals in Xi’an City, Northwest China	2014–2016	474	948	Dietary assessment based on the FFQ items	Heavy metals	“Mothers whose fetuses were diagnosed with isolated CHDs and had no chromosomal abnormalities or gene disorders were included in the cases”.	“The significantly inverse associations with CHDs were also observed for dietary intakes of zinc to copper ratio, selenium to copper ratio, and zinc and selenium supplements use during pregnancy and in the first trimester. Moreover, high zinc and high selenium, even with low or high copper, showed a significantly reduced risk of total CHDs. Efforts to promote zinc and selenium intakes during pregnancy need to be strengthened to reduce the incidence of CHDs in the Chinese population”.
Zhang 2017 [73]	China	Case–control	-	2010–2011	399	490	Hair sample, fetal placental tissue	Heavy metals	“Fetuses diagnosed with cardiac defects prenatally were recruited as the cases”.	“Maternal barium exposure was dose-dependently related to the risk of CHD in the offspring. Our findings suggest that the occurrence of some subtypes of CHD is associated with barium exposure”.
Zhang 2019 [74]	China	Case–control	Five maternal and child hospitals cities of Zhengzhou, Shenzhen, Fuzhou, Xi’an, and Wuhan in China	2010–2013	399	490	Hair sample, fetal placental tissue	Heavy metals	“Fetuses diagnosed with cardiac defects prenatally were recruited as the cases”.	“The finding suggested that the occurrence of CHDs may be associated with nickel exposure”.
Zhang 2020 [75]	China	Case–control	Five maternal and child hospitals cities of Zhengzhou, Shenzhen, Fuzhou, Xi’an, and Wuhan in China	2010–2013	399	490	Hair sample, fetal placental tissue	Heavy metals	“Fetuses diagnosed with cardiac defects prenatally were recruited as the cases”	“The finding suggested that the occurrence of CHDs may be associated with cobalt exposure”.
Zhang 2022 [76]	China	Cohort	Guangz houPanyu Maternal Child Health Hospital	2011–2014	39	3928	Job–exposure matrix	EDCs	“All the CHD defects were confirmed by using B-mode echocardiography, cardiac catheterization, surgery, or autopsy. They were coded by using a modified code from the International Classification of Diseases (ICD), 10th Revision (Q20.000–Q28.000) and classified according to the criteria of US NBDPS”.	“Maternal exposure to EDCs was associated with greater odds of birth defects and CHD, while paternal exposure was mainly associated with greater odds of LBW. These effects tend to be stronger among mothers without multi-vitamin supplements and among male babies”.
Zierler 1988 [77]	USA	Case–control	-	1980–1983	440	929	Maternal residential address proximity to corresponding water supply	Heavy metals	-	“A dose–response effect was observed over four levels of selenium exposure. Non-differential errors in measuring and classifying exposure to contaminants routinely monitored in drinking water could account for the lack of positive findings. In addition, most of the contaminant levels were below the maximum levels set by the Environmental Protection Agency, so that lack of evidence of effect may have been due to the low exposure levels in this population”.

Furthermore, maternal exposure to heavy metals, phthalates, APCs, BPAs, PCBs, pesticides, PAHs, and solvents did not reveal any notable relationship with the development of CHDs (O.R. = 0.90, 95% C.I. [0.68–1.18], *p* = 0.43), (O.R. = 1.18, 95% C.I. [0.67–2.07], *p* = 0.57), (O.R. = 1.36, 95% C.I. [0.88–2.12], *p* = 0.17), (O.R. = 0.99, 95% C.I. [0.52–1.86], *p* = 0.97), (O.R. = 1.19, 95% C.I. [0.74–1.92], *p* = 0.47), (O.R. = 1.04, 95% C.I. [0.64–1.67], *p* = 0.88), (O.R. = 1.01, 95% C.I. (0.89–1.14), *p* = 0.92), and (O.R. = 1.12, 95% C.I. [0.90–1.39], *p* = 0.32). However, housing renovation compounds were correlated to a heightened incidence of CHDs (O.R. = 1.56, 95% C.I. [1.14–2.14], *p* = 0.006), as illustrated in Figure 2.

**Figure 3:**
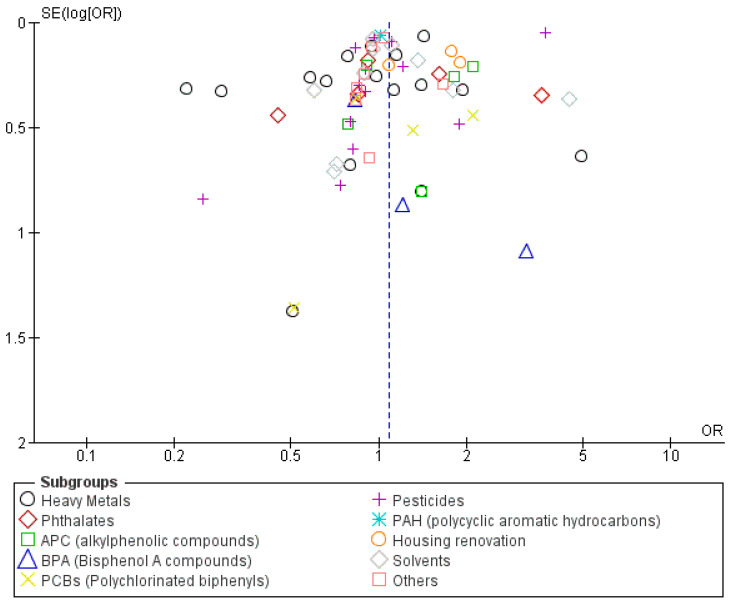
Funnel plot of publication bias for congenital heart defect incidence measured by non-sample methods. This funnel plot assesses the potential publication bias in studies examining the incidence of congenital heart defects measured by non-sample methods. Symmetry in the plot suggests the absence of publication bias.

A funnel plot was constructed, and upon examination, it showed a symmetrical pattern, suggesting the possible existence of publication bias, as illustrated in Figure 3.

**Figure 4:**
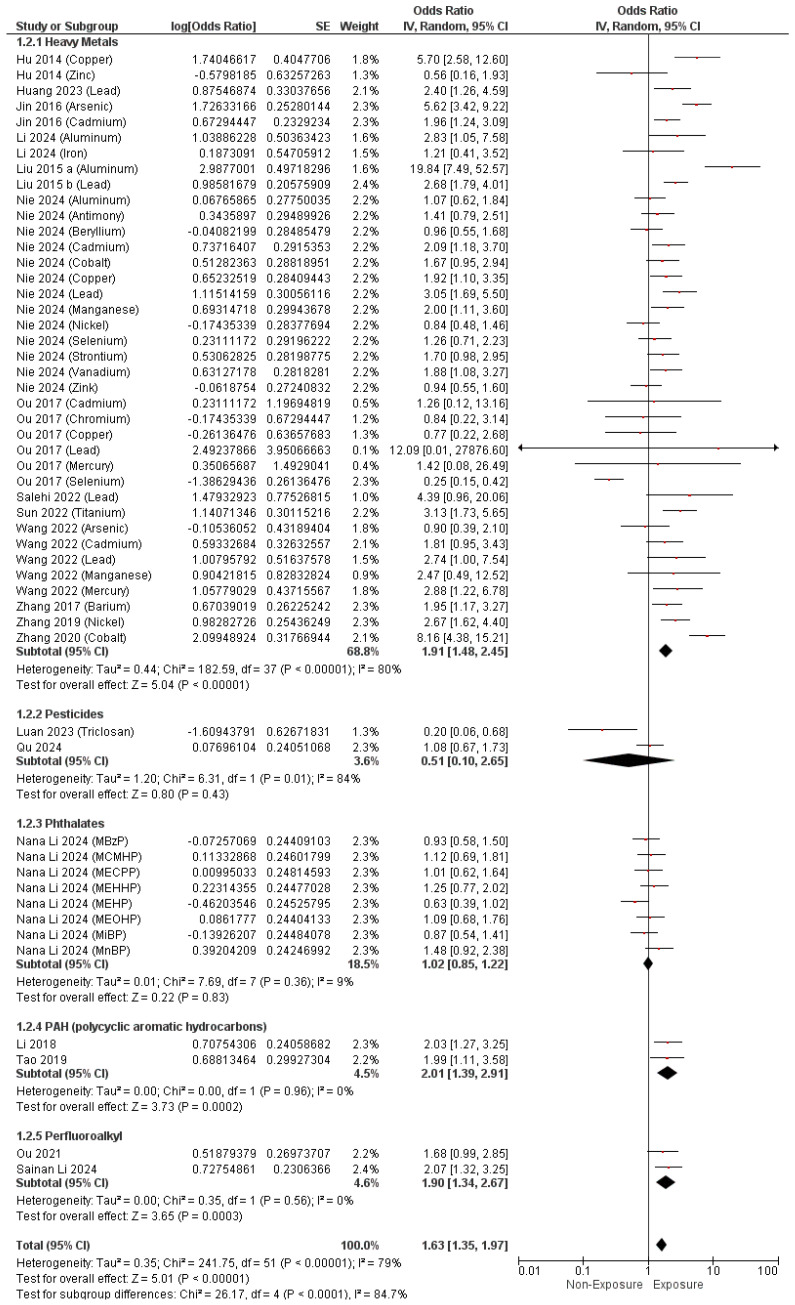
Forest plot of congenital heart defect incidence measured using human samples. This forest plot presents the odds ratios and 95% confidence intervals for the association between maternal exposure to endocrine-disrupting chemicals and the incidence of congenital heart defects, based on human sample methods of exposure assessment. Subgroups include heavy metals, polycyclic aromatic hydrocarbons, perfluoroalkyl compounds, pesticides, and phthalates.

Regarding EDCs measured using human samples, the pooled analysis revealed a notable link between maternal exposure to EDCs and the development of CHDs with a combined O.R. of 1.63 (95% C.I. [1.35–1.97], *p* < 0.00001). The pooled results demonstrated notable heterogeneity (I^2^ = 79%, *p* < 0.00001), as presented in Figure 4.

Additionally, maternal exposure to heavy metals (O.R. = 1.91, 95% C.I. [1.48–2.45], *p* < 0.00001), PAHs (O.R. = 2.01, 95% C.I. [1.39–2.91], *p* = 0.0002), and perfluoroalkyl (O.R. = 1.90, 95% C.I. [1.34–2.67], *p* = 0.0003). Nevertheless, pesticides (O.R. = 0.51, 95% C.I. [0.10–2.65], *p* = 0.43) and phthalates (O.R. = 1.02, 95% C.I. [0.85–1.22], *p* = 0.83) exposure did not reveal any significant association with the development of CHDs, as illustrated in Figure 4.

**Figure 5:**
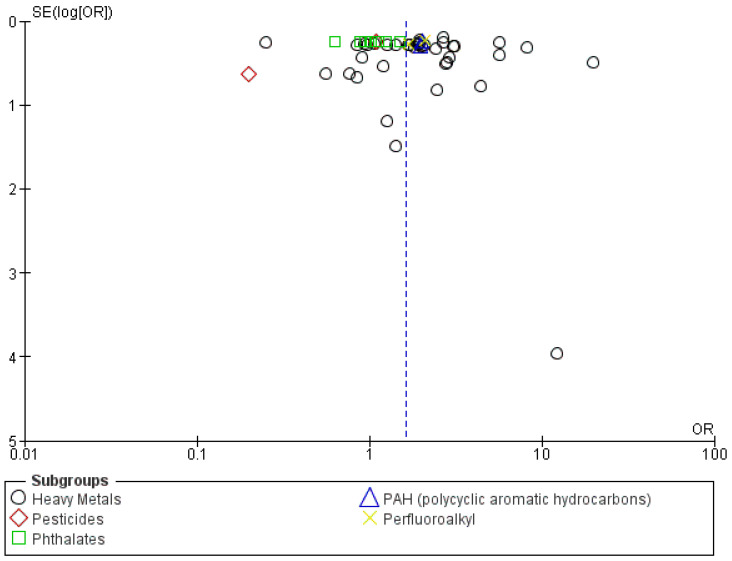
Funnel plot of publication bias for congenital heart defect incidence measured using human samples. This funnel plot evaluates the potential publication bias in studies examining the incidence of congenital heart defects measured using human samples. An asymmetrical pattern indicates possible publication bias.

There is a possible publication bias based on the asymmetrical pattern observed on the funnel plot, as shown in Figure 5.

#### 3.4.2. Septal Heart Defects

**Figure 6:**
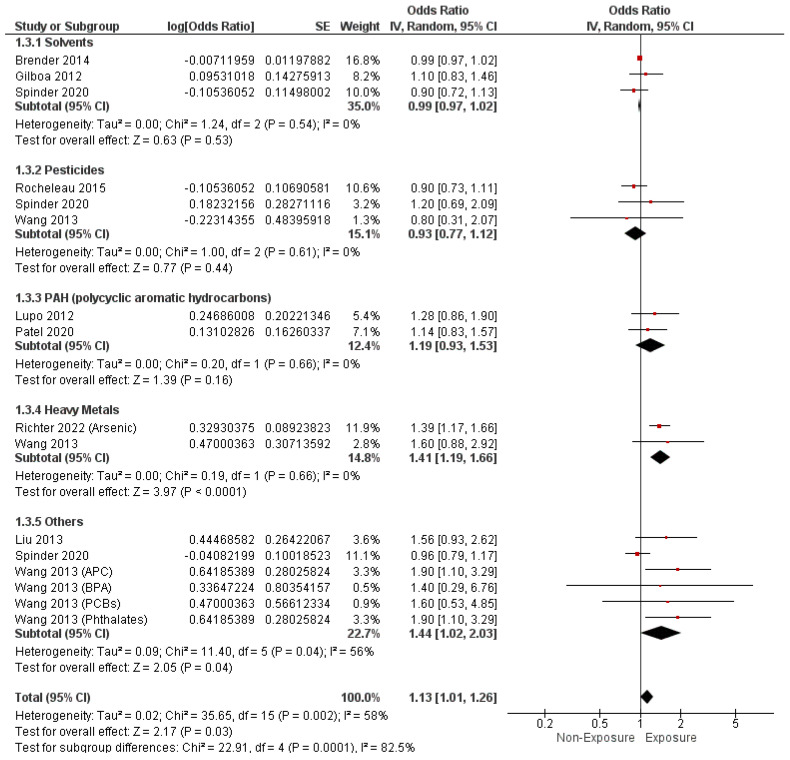
Forest plot of septal heart defect incidence measured by non-sample methods. This forest plot displays the odds ratios and 95% confidence intervals for the association between maternal exposure to endocrine-disrupting chemicals and the incidence of septal heart defects, based on non-sample methods of exposure assessment. Subgroups include heavy metals, pesticides, solvents, and polycyclic aromatic hydrocarbons.

Regarding EDCs measured by non-sample methods, the overall OR showed a substantial correlation between maternal exposure to EDCs and the incidence of septal heart defects (O.R. = 1.13, 95% C.I. [1.01–1.26], *p* = 0.03). Combined results showed notable heterogeneity (I^2^ = 58%, *p* = 0.002), as presented in Figure 6.

Conversely, maternal exposure solvents (O.R. = 0.99, 95% C.I. [0.97–1.02], *p* = 0.53), pesticides (O.R. = 0.93, 95% C.I. [0.77–1.12], *p* = 0.44), and PAHs (O.R. = 1.19, 95% C.I. [0.93–1.53], *p* = 0.16) were not related to a notable risk of septal heart defects. However, maternal exposure to heavy metals showed a higher likelihood of developing septal heart defects (O.R. = 1.41, 95% C.I. [1.19–1.66], *p* < 0.0001), as presented in Figure 6.

**Figure 7:**
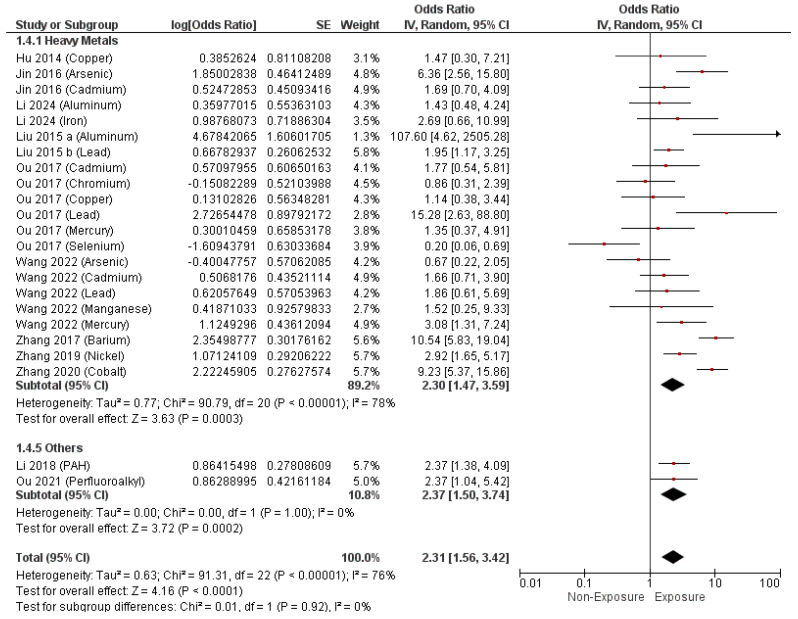
Forest plot of septal heart defect incidence measured using human samples. This forest plot shows the odds ratios and 95% confidence intervals for the association between maternal exposure to endocrine-disrupting chemicals and the incidence of septal heart defects, based on human sample methods of exposure assessment. Subgroups include heavy metals and polycyclic aromatic hydrocarbons.

Regarding EDCs measured by sample methods, the pooled analysis revealed a significant correlation between maternal exposure to EDCs and the development of septal heart defects (O.R. = 2.31, 95% C.I. [1.56–3.42], *p* < 0.0001) with a substantial heterogeneity (I^2^ = 76%, *p* < 0.00001), as shown in Figure 7. Furthermore, mothers exposed to heavy metals showed a higher risk of developing septal heart defects (O.R. = 2.30, 95% C.I. [1.47–3.59], *p* = 0.0003), as illustrated in Figure 7.

#### 3.4.3. Ventricular Septal Defects

**Figure 8:**
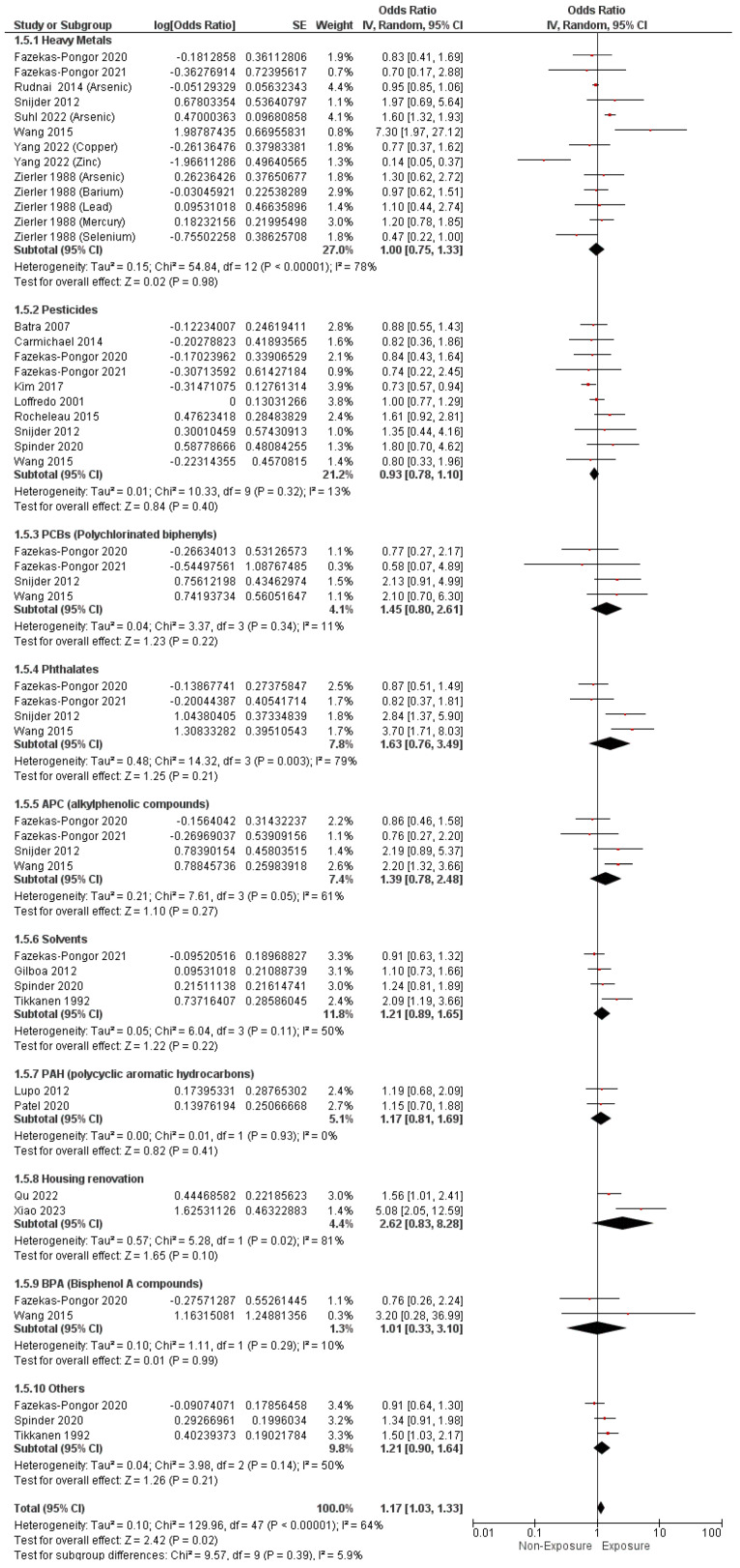
Forest plot of ventricular septal defect incidence measured by non-sample methods. This forest plot presents the odds ratios and 95% confidence intervals for the association between maternal exposure to endocrine-disrupting chemicals and the incidence of ventricular septal defects, based on non-sample methods of exposure assessment. Subgroups include heavy metals, pesticides, polychlorinated biphenyls, phthalates, alkylphenolic compounds, solvents, polycyclic aromatic hydrocarbons, housing renovation compounds, and bisphenol A.

Based on the EDCs measured by the non-sample methods, the pooled OR showed a notable correlation between maternal exposure to EDCs and the incidence of VSDs with a pooled O.R. of 1.17 (95% C.I. [1.03–1.33], *p* = 0.02) with an observed substantial heterogeneity (I^2^ = 64%, *p* < 0.00001), as shown in Figure 8. Nevertheless, maternal exposure to heavy metals (O.R. = 1, 95% C.I. [0.75–1.33], *p* = 0.98), pesticides (O.R. = 0.93, 95% C.I. [0.78–1.10], *p* = 0.40), PCBs (O.R. = 1.45, 95% C.I. [0.80–2.61] *p* = 0.22), phthalates (O.R. = 1.63, 95% C.I. [0.76–3.49], *p* = 0.21), APCs (O.R. = 1.39, 95% C.I. (0.78–2.48], *p* = 0.27), solvents (O.R. = 1.21, 95% C.I. [0.89–1.65], *p* = 0.22), PAHs (O.R. = 1.17, 95% C.I. [0.81–1.69], *p* = 0.41), housing renovation compounds (O.R. = 2.62, 95% C.I. [0.83–8.28], *p* = 0.10), and BPAs (O.R. = 1.01, 95% C.I. [0.33–3.10], *p* = 0.99) revealed a correlation, as illustrated in Figure 8.

#### 3.4.4. Atrial Septal Defects

Utilizing the non-sample methods of measuring the EDCs, the overall OR did not reveal any notable correlation between maternal exposure to EDCs and the occurrence of ASDs (O.R. = 1.01, 95% C.I. [0.78–1.30], *p* = 0.94). The combined results were associated with notable heterogeneity (I^2^ = 92%, *p* < 0.00001), as illustrated in Supplementary Figure S1.

Additionally, there was no substantial correlation between maternal exposure to pesticides (O.R. = 1.13, 95% C.I. [0.58–2.20], *p* = 0.73), heavy metals (O.R. = 0.75, 95% C.I. [0.46–1.23], *p* = 0.25), solvents (O.R. = 1.06, 95% C.I. [0.85–1.32], *p* = 0.61), PCBs (O.R. = 1.01, 95% C.I. [0.46–2.23], *p* = 0.98), BPAs (O.R. = 0.89, 95% C.I. [0.34–2.34], *p* = 0.81), phthalates (O.R. = 1.20, 95% C.I. [0.61–2.37], *p* = 0.60), APCs (O.R. = 1.00, 95% C.I. [0.65–1.55], *p* = 0.99), PAHs (O.R. = 0.84, 95% C.I. [0.49–1.42], *p* = 0.51), housing renovation compounds (O.R. = 1.27, 95% C.I. [0.71–2.25], *p* = 0.42) and the risk of ASDs, as illustrated in Supplementary Figure S1.

#### 3.4.5. Conotruncal Heart Defects

Utilizing non-sample methods of measuring EDCs, maternal exposure to EDCs was not correlated with an elevated risk of conotruncal heart defects (O.R. = 1.09, 95% C.I. [0.96–1.24], *p* = 0.18) with an observed significant heterogeneity (I^2^ = 45%, *p* = 0.03), as shown in Supplementary Figure S2.

Maternal exposure to heavy metals (O.R. = 0.89, 95% C.I. [0.71–1.11], *p* = 0.29), pesticides (O.R. = 0.98, 95% C.I. [0.75–1.29], *p* = 0.88), and solvents (O.R. = 1.18, 95% [0.88–1.59] *p* = 0.28) did not reveal any significant correlation with the development of conotruncal heart defects. However, maternal exposure to PAHs showed an elevated risk of developing conotruncal heart defects (O.R. = 1.45, 95% C.I. [1.07–1.95], *p* = 0.02), as exhibited in Supplementary Figure S2.

Utilizing sample methods of measuring EDCs, the pooled analysis revealed a notable relationship between maternal exposure to EDCs and the development of conotruncal heart defects (O.R. = 2.61, 95% C.I. [1.72–3.57], *p* < 0.00001). The pooled results showed substantial heterogeneity (I^2^ = 77%, *p* < 0.00001), as presented in Supplementary Figure S3. Additionally, mothers exposed to heavy metals showed a higher risk of bearing children with conotruncal heart defects (O.R. = 2.77, 95% C.I. [1.74–4.39], *p* < 0.0001), as illustrated in Supplementary Figure S3.

#### 3.4.6. Tetralogy of Fallot

Concerning EDCs measured by non-human sample methods, maternal exposure to EDCs was associated with an elevated risk of tetralogy of Fallot (O.R. = 1.34, 95% C.I. [1.11–1.61], *p* = 0.002) with corresponding significant heterogeneity (I^2^ = 39%, *p* = 0.06), as pointed out in Supplementary Figure S4.

Maternal exposure to pesticides (O.R. = 1.16, 95% C.I. [0.83–1.61], *p* = 0.39), heavy metals (O.R. = 1.40, 95% C.I. [0.56–3.53], *p* = 0.47), and solvents (O.R. = 1.11, 95% C.I. [0.73–1.68], *p* = 0.63) was not associated with an elevated risk of tetralogy of Fallot, as illustrated in Supplementary Figure S4.

Nevertheless, an elevated incidence of tetralogy of Fallot was noted within mothers exposed to PAHs (O.R. = 1.74, 95% C.I. [1.23–2.45], *p* = 0.002), as pointed out in Supplementary Figure S4.

#### 3.4.7. Transposition of Great Arteries

Maternal exposure to EDCs revealed a heightened risk of bearing children with the transposition of great arteries based on the non-sample methods (O.R. = 1.26, 95% C.I. [1.04–1.52], *p* = 0.02) with corresponding non-significant heterogeneity (I^2^ = 20%, *p* = 0.20), as illustrated in Supplementary Figure S5.

Pesticide exposure was associated with a heightened risk of developing transposition of great arteries (O.R. = 1.83, 95% C.I. [1.22–2.74], *p* = 0.004).

However, the maternal exposure to heavy metals (O.R. = 0.89, 95% C.I. [0.71–1.11], *p* = 0.30), solvents (O.R. = 1.24, 95% C.I. [0.86–1.77], *p* = 0.25), PCBs (O.R. = 1.42, 95% C.I. [0.44–4.63], *p* = 0.56), APCs (O.R. = 1.50, 95% C.I. [0.49–4.57], *p* = 0.48), phthalates (O.R. = 1.76, 95% C.I. [0.79–3.90], *p* = 0.17), was not associated with a heightened risk, as shown in Supplementary Figure S5.

#### 3.4.8. Left Ventricular Outflow Tract Obstruction Defects

Using the non-sample methods for measuring EDCs, the pooled analysis did not reveal any significant association between maternal exposure to EDCs and the incidence of LVOTODs with a pooled O.R. of 1.01, (95% C.I. [0.88–1.15], *p* = 0.92) with non-statistically significant heterogeneity (I^2^ = 0%, *p* = 0.58), as presented in Supplementary Figure S6.

Furthermore, maternal exposure to pesticides (O.R. = 1.04, 95% C.I. [0.85–1.26], *p* = 0.73), solvents (O.R. = 0.89, 95% C.I. [0.68–1.16], *p* = 0.38), phthalates (O.R. = 1.18, 95% C.I. [0.10–13.58], *p* = 0.90), and PAHs (O.R. = 1.27, 95% C.I. [0.91–1.78], *p* = 0.16) did not show any significant relationship with the development of LVOTODs, as shown in Supplementary Figure S6.

Using the sample methods for measuring the EDCs, the pooled analysis revealed a significant relationship between maternal exposure to EDCs and bearing a child with LVOTODs (O.R. = 4.00, 95% C.I. [2.65–6.03], *p* < 0.00001), as presented in Supplementary Figure S7. Interestingly, mothers exposed to heavy metals (O.R. = 4.34, 95% C.I. [2.73–6.90], *p* < 0.00001) and PAHs (O.R. = 2.66, 95% C.I. [1.09–6.53], *p* = 0.03) showed an elevated risk of bearing children with LVOTODs, as illustrated in Supplementary Figure S7.

#### 3.4.9. Hypoplastic Left Heart Syndrome

Based on the non-sample methods of measuring EDCs, maternal exposure to EDCs did not show any notable association with the development of hypoplastic left heart syndrome (O.R. = 1.33, 95% C.I. [0.83–2.13], *p* = 0.24) with associated significant heterogeneity (I^2^ = 90%, *p* < 0.00001), as presented in Supplementary Figure S8.

Maternal exposure to solvents (O.R. = 0.94, 95% C.I. [0.40–2.22], *p* = 0.89), pesticides (O.R. = 2.15, 95% C.I. [0.94–4.90], *p* = 0.07), and PAHs (O.R. = 1.17, 95% C.I. [0.70–1.94], *p* = 0.55) were not notably related to an elevated risk of hypoplastic left heart syndrome, as illustrated in Supplementary Figure S8.

#### 3.4.10. Coarctation of Aorta

According to the non-sample methods of measuring EDCs, the overall OR did not reveal any substantial linkage between maternal exposure to EDCs and the incidence coarctation of the aorta (O.R. = 1.23, 95% C.I. [0.95–1.59], *p* = 0.11), with correlated notable heterogeneity (I^2^ = 55%, *p* = 0.002), as shown in Supplementary Figure S9.

Moreover, the statistically in-significant OR did not change notably in regard to the maternal exposure to heavy metals (O.R. = 1.01, 95% C.I. [0.58–1.74], *p* = 0.98), solvents (O.R. = 1.05, 95% C.I. [0.68–1.62], *p* = 0.83), pesticides (O.R. = 1.40, 95% C.I. [0.65–3.00], *p* = 0.38), and PAHs (O.R. = 1.55, 95% C.I. [0.93–2.58], *p* = 0.09), as illustrated in Supplementary Figure S9.

#### 3.4.11. Aortic Stenosis

Maternal exposure to EDCs was not significantly related to a heightened risk of aortic stenosis based on the non-sample methods for measuring EDCs (O.R. = 1.01, 95% C.I. [0.56–1.83], *p* = 0.97). Combined studies showed significant heterogeneity (I^2^ = 67%, *p* = 0.03), as presented in Supplementary Figure S10.

#### 3.4.12. Right Ventricular Outflow Tract Obstruction Defects

Utilizing the non-sample methods in measuring the EDCs, the pooled OR did not demonstrate any significant correlation between maternal exposure to EDCs and the incidence of RVOTODs (O.R. = 1.04, 95% C.I. [0.88–1.22], *p* = 0.67) linked to a non-substantial heterogeneity (I^2^ = 14%, *p* = 0.28), as pointed out in Supplementary Figure S11.

Additionally, OR did not significantly change from the pooled OR regarding the maternal exposure to pesticides (O.R. = 0.99, 95% C.I. [0.65–1.50], *p* = 0.96), solvents (O.R. = 1.19, 95% C.I. [0.92–1.54], *p* = 0.18), APCs (O.R. = 0.35, 95% C.I. [0.10–1.22], *p* = 0.10), and phthalates (O.R. = 1.23, 95% C.I. [0.59–2.56], *p* = 0.58), as illustrated in Supplementary Figure S11.

However, maternal exposure to PAHs was linked to a reduced risk of RVOTODs (O.R. = 0.61, 95% C.I. [0.38–0.97], *p* = 0.04), as illustrated in Supplementary Figure S11.

Based on measuring the EDCs using sample methods, the pooled OR revealed a significant relationship between maternal exposure to EDCs and the risk of developing RVOTODs (O.R. = 3.11, 95% C.I. [1.65–5.88], *p* = 0.0005) with associated significant heterogeneity (I^2^ = 78%, *p* < 0.00001), as shown in Supplementary Figure S12. Additionally, the overall OR remained statistically significant regarding the maternal exposure to heavy metals (O.R. = 3.17, 95% C.I. [1.56–6.44], *p* = 0.001) and PAHs (O.R. = 2.42, 95% C.I. [1.19–4.93], *p* = 0.01), as shown in Supplementary Figure S12.

#### 3.4.13. Pulmonary Valve Stenosis

According to the non-sample methods of measuring EDCs, maternal exposure to EDCs was linked to a heightened risk of pulmonary valve stenosis (O.R. = 1.33, 95% C.I. [1.03–1.73], *p* = 0.03) with corresponding significant heterogeneity (I^2^ = 62%, *p* = 0.001), as pointed out in Supplementary Figure S13.

Nonetheless, maternal exposure to pesticides (O.R. = 1.24, 95% C.I. [0.88–1.75], *p* = 0.21), heavy metals (O.R. = 1.08, 95% C.I. [0.31–3.74], *p* = 0.90), and solvents (O.R. = 1.22, 95% C.I. [0.88–1.68], *p* = 0.23), was not linked to a heightened risk, as illustrated in Supplementary Figure S13.

#### 3.4.14. Patent Ductus Arteriosus

Based on the non-sample methods of measuring EDCs, the overall OR demonstrated that maternal exposure to EDCs was linked to an elevated risk of PDA (O.R. = 1.68, 95% C.I. [1.25–2.26], *p* = 0.0005) with correlated significant heterogeneity (I^2^ = 71%, *p* < 0.00001), as presented in Supplementary Figure S14.

The pooled OR did not change significantly regarding maternal exposure to pesticides (O.R. = 2.44, 95% C.I. [1.47–4.05], *p* = 0.0005), APCs (O.R. = 1.89, 95% C.I. [1.19–3.00], *p* = 0.007), and housing renovation compounds (O.R. = 2.34, 95% C.I. [1.34–4.08], *p* = 0.003), as illustrated in Supplementary Figure S14.

However, the pooled analysis did not show any relationship with PDA concerning maternal exposure to heavy metals (O.R. = 1.21, 95% C.I. [0.92–1.59], *p* = 0.18), phthalates (O.R. = 1.57, 95% C.I. [0.61–4.06], *p* = 0.35), BPAs (O.R. = 2.49, 95% C.I. [0.64–9.67], *p* = 0.19), and PCBs (O.R. = 1.85, 95% C.I. [0.70–4.90], *p* = 0.21), as shown in Supplementary Figure S14.

Based on the sample methods for measuring EDCs, the pooled analysis revealed a notable relationship between maternal exposure to EDCs, particularly heavy metals, and the risk of developing PDA (O.R. = 2.23, 95% C.I. [1.35–3.67], *p* = 0.002) with corresponding non-statistically substantial heterogeneity (I^2^ = 0%, *p* = 0.97), as shown in Supplementary Figure S15.

#### 3.4.15. Total Anomaly Pulmonary Venous Return

Utilizing non-sample methods for measuring EDCs, mothers exposed to EDCs did not show any significant risk of bearing child with TAPVR (O.R. = 1.62, 95% C.I. [0.94–2.79], *p* = 0.08). Combined studies revealed substantial heterogeneity (I^2^ = 69%, *p* = 0.003), as illustrated in Supplementary Figure S16.

Based on the sample methods for measuring EDCs, maternal exposure to EDCs was associated with a heightened risk of TAPVR (O.R. = 3.56, 95% C.I. [1.80–7.05], *p* = 0.0003) with related significant homogeneity (I^2^ = 43%, *p* = 0.16), as pointed out in Supplementary Figure S17.

## 4. Discussion

Utilizing the non-sample methods for measuring EDCs, the pooled OR showed maternal exposure to EDCs had no significant association with CHDs. Interestingly, only mothers exposed to housing renovation compounds showed higher odds of bearing a child with CHD. Septal heart defects were significantly related to maternal exposure to EDCs, particularly heavy metals, which showed a significant association with septal heart defects. Furthermore, maternal exposure to EDCs was significantly related to an elevated risk of VSDs. Nevertheless, ASDs were not notably related to maternal exposure to EDCs. Maternal exposure to EDCs in-significantly showed an elevated risk of conotruncal heart defects. Only PAHs were notably linked to a higher risk of conotruncal heart defects. Mothers exposed to EDCs substantially showed an elevated risk of bearing a child with tetralogy of Fallot, especially PAH compounds. Additionally, TGAs showed a statistically significant relationship with maternal exposure to EDCs. Only pesticides were notably correlated with an elevated risk of TGAs. Conversely, maternal exposure to EDCs was not significantly correlated with a heightened risk of LVOTODs. Also, the overall OR did not reveal any significant relationship between maternal exposure to EDCs and hypoplastic left heart syndrome. Moreover, RVOTODs were not notably correlated with maternal exposure to EDCs. Conversely, maternal exposure to EDCs showed an elevated risk of pulmonary valve stenosis. Also, PDA was substantially associated with maternal exposure to EDCs, particularly pesticides, APCs, and housing renovation compounds. Finally, maternal exposure to EDCs was not notably correlated with an elevated risk of TAPVR. Utilizing sample methods for measuring EDCs, the pooled OR demonstrated a substantial relationship between maternal exposure to EDCs and CHDs, especially heavy metals, PAHs, and perfluoroalkyl compounds. Septal heart defects were notably associated with maternal exposure to EDCs, especially heavy metals. Also, conotruncal heart defects revealed a notable relationship regarding maternal exposure to EDCs. Finally, maternal exposure to EDCs was statistically significantly associated with LVOTODs, RVOTODs, PDAs, or TAPVRs (Table 2).

Even with significant advancements in diagnosis and management, CHDs are still a significant cause of mortality among infants [78]. Although children affected by CHD may live into adulthood, they face a heightened risk of mortality and morbidity due to late complications such as heart failure and arrhythmia [79]. CHDs result from the interaction of genetic and environmental factors. While genetic factors are fixed, environmental factors can be modified through appropriate intervention [80]. Therefore, ensuring maternal care during pregnancy is crucial to mitigate the risk of CHDs in offspring. Exposure to harmful environmental substances during pregnancy can induce mutagenic and teratogenic outcomes, resulting in congenital defects [81]. Many environmental factors, known as EDCs, have been identified, including heavy metals, pesticides, phthalates, PAHs, APCs, and other compounds [2].

In their recently published meta-analysis, Dai and his colleagues examined the relationship between maternal exposure to EDCs and the incidence of CHDs [10]. They only included studies that measured the EDC concentration using human biospecimens such as blood, urine, and hair specimens. With 17 studies included in their research, they identified a substantial relationship between maternal exposure to EDCs and the incidence of CHDs, which is aligned with our study findings concerning EDCs measured using sample methods. Moreover, after they grouped analyses based on CHD categories, they also identified a notable relationship between EDCs and conotruncal heart defects, RVOTODs, LVOTODs, and other heart defects. Their subgroup analysis based on EDC type, especially heavy metals, showed a substantial association between maternal exposure to heavy metals, including lead, cadmium, mercury, and manganese, and the risk of CHDs. Our results based on sample methods of measurement were aligned with their results. The underlying mechanisms for CHDs with heavy metals exposure include decreased antioxidant capacity, oxidative stress, myocardial mitochondrial dysfunction, and abnormal homocysteine levels [48,82,83]. Exposure to heavy metals could also damage the chromosomes and DNA of germ cells, potentially influencing embryo implantation and development [84]. Additionally, an imbalance in reproductive hormones caused by heavy metal exposure could be linked to birth anomalies [85].

Our results depended on the non-sample methods of measuring EDCs and showed a non-statistically significant relationship between heavy metals exposure and the risk of CHDs. Snijder et al. [59] conducted a case–control study with 424 mothers and 421 fathers of CHD children and 480 mothers and 477 fathers of non-diseased children. Mothers and fathers who participated in this study completed a standardized questionnaire regarding pre-conceptional general and job features. Then, a job exposure matrix integrating work tasks and job titles with expert judgment was applied to assess occupational exposure to chemicals. For included mothers, the prevalence of occupational exposure to chemicals was 5% for cases and 6.2% for controls. Moreover, they did not find any significant relationship between maternal exposure to heavy metals and the risk of CHDs, which aligned with our findings. This finding could be explained using a job–exposure matrix for exposure assessment, as it did not account for variability within the different job titles. It also depends on a questionnaire completed by mothers asking them about their pre-conceptional job characteristics, which potentially may introduce some recall bias. Also, the observational nature of this study could introduce some selection bias.

Conversely, a nationwide cohort study executed by Richter [54] and his colleagues concluded that there is a notable relationship between maternal exposure to heavy metals, particularly arsenic, and CHDs and septal heart defects. However, they depend on the exposure assessment of arsenic in residential proximity to the sources of drinking water, which could be influenced by mothers drinking from another water supply area. Mothers may not be linked to a public water supply and may live at different locations with different arsenic levels during embryology. As a result, the yearly mean arsenic concentration would differ from the true exposure level during this timeframe.

Pesticides are one of the most prevalent EDCs, and they have been shown to disrupt the development and growth in experimental animals and potentially in humans [59]. Based on the non-sample methods of measuring EDCs, our results did not show any significant association between maternal exposure to pesticides and CHDs. In their case–control study, Fazekas-Pongor and his colleagues did not find any notable relationship between maternal exposure to pesticides and the occurrence of CHDs and its subtypes, encompassing VSD, ASD, RVOTODs, LVOTODs, PDA, and TGA [27]. A possible explanation for their findings is that they applied a job–exposure matrix to evaluate occupational exposure to chemicals. This job–exposure matrix lacks validation with actual measurement data. Also, this job–exposure matrix combines pesticides and heavy metals into a single group, hindering the ability to separate the effects of individual compounds.

Our primary analysis based on sample measurement methods did not show any substantial correlation between maternal exposure to pesticides and the incidence of CHDs. This could be explained by the fewer studies (Two) that measured the pesticides using human samples.

Li et al. [37] executed a case–control study, with a total of 357 mothers of CHD cases and 270 control mothers, to assess the correlation between maternal PAH exposure and CHDs. They measured the exposure to PAHs utilizing urine specimens. With the low concentration of PAH exposure as a reference, they found a substantial correlation between maternal exposure to PAHs and CHDs and its subtypes involving septal heart defects, conotruncal heart defects, RVOTODs, LVOTODs, and TAPVR, which is agreed with our findings based on samples as methods of measuring EDCs exposure. However, our results based on non-sample methods showed a non-significant relationship between maternal PAH exposure and the risk of CHDs, which was aligned with Patel et al. [50]’s investigation. PAHs are prevalent in the environment and initially originate from coal, gasoline, and diesel emissions. The increasing number of vehicles heightens the risk of population exposure to PAHs [86]. Benzo (a) pyrene (BaP), which is a typical PAH, can have adverse effects on the female reproductive system [87]. Animal research demonstrated that BaP exposure during pregnancy in mice disrupted the imbalance between estrogen and progesterone values, impacting receptor expression and corresponding gene expression, which consequently leads to changes in endometrial tolerance and reduces the implantation sites [88]. Furthermore, BaP could trigger oxidative stress by activating the aryl hydrocarbon receptor, resulting in mitochondrial-mediated intrinsic apoptosis and inducing cardiac anomalies in zebrafish embryos [89].

Phthalates, a well-known EDC, exhibit high lipophilicity and are rapidly taken up by humans and animals [90]. Our results based on the non-sample methods did not reveal any notable correlation between maternal exposure to phthalates and the risk of CHDs. These findings were aligned with those of the study by Fazekas-Pongor et al. [27]. Utilizing a job–exposure matrix for assessing occupational exposure to EDCs, Wang et al. [2] found a notable relationship between maternal phthalate exposure and a higher incidence of CHDs. These significant findings could be attributed to the fact that this study was conducted in China, particularly in the southwestern part of China, which has a low economic level. These low economic levels necessitated more women working outside the home to earn enough money. Nevertheless, most of them had low education levels. So, most of them were limited to manual work in factories, resulting in more chances of chemical exposure.

Utilizing the sample methods for measuring phthalates, no significant relationship between maternal exposure to phthalates and the risk of CHDs was noticed. In their multicenter case–control study, Li and his colleagues examined the association between maternal exposure to phthalates, using maternal urine samples for exposure assessment, and the risk of CHDs [45]. There was no notable correlation between phthalate exposure and CHDs, which aligns with our investigation. This finding could be attributed to the small sample size, which may limit the statistical power.

Our primary analysis revealed a significant correlation between maternal exposure to housing renovation compounds and the risk of CHDs based on the non-sample methods of measuring housing renovation substances. This was aligned with a multi-hospital case–control investigation executed by Liu et al. and performed within the Chinese population [38].

Additionally, maternal exposure to solvents did not reveal any substantial relationship with the incidence of CHDs. Motoki et al. [44] carried out an observational study utilizing data from the national birth cohort study. They implemented multiple logistic regression analyses to examine the association between maternal exposure to solvents and the risk of CHDs. They also did not find any notable relationship between maternal exposure to solvents and the risk of CHDs. However, their findings were limited by their use of self-reported questionnaires as methods for exposure assessment. 

In a recent study by Kai Pan et al. (2024) published in Environmental Research Letters, the authors conducted a systematic review and meta-analysis to investigate the association between maternal exposure to EDCs and the risk of CHDs in offspring [91]. Their analysis included 17 studies with a total of 137,311 participants. While their findings align with our conclusions regarding the significant impact of EDCs on CHDs, our study offers several unique contributions. We included a more extensive dataset comprising 59 studies with a total of 1,373,117 participants, enhancing our results’ statistical power and generalizability. Additionally, our comprehensive approach encompassed all available EDCs in the literature without restricting the analysis to specific chemicals, providing a broader understanding of the potential impact of various EDCs on CHDs. Furthermore, our detailed subgroup analyses based on different types of EDCs and exposure assessment methods offer more nuanced insights into the specific effects of various EDCs on CHDs, distinguishing our study from previous research. These aspects underscore the robustness and uniqueness of our findings, contributing valuable insights to the ongoing conversation in public health and environmental science.

While our primary focus was on endocrine-disrupting chemicals, it is important to acknowledge that not all chemicals included in this analysis exert their effects through endocrine disruption. For instance, metals such as lead and cadmium may contribute to congenital heart diseases through mechanisms such as oxidative stress, mitochondrial dysfunction, and genetic damage. These alternative pathways should be considered in future research to fully understand the multifaceted nature of chemical exposures and their impacts on fetal development.

Our investigation possesses several strengths. First, to the best of our knowledge, our study provides the most comprehensive updated evidence regarding the link between maternal exposure to EDCs and the risk of CHDs, with 59 studies ultimately included. Second, the studies incorporated in our meta-analysis were conducted in different geographical regions within different populations. Third, the quality of the included studies ranged from good to moderate quality, thus increasing the chance of generalizability and applicability of our findings.

Nevertheless, our study has multiple limitations. First, substantial heterogeneity was observed between the retrieved studies in the majority of endpoints. This can be attributed to the variability in assessed chemicals, populations studied, and exposure assessment methods. Second, the case–control observational design of the majority of the included studies could introduce some selection bias. Third, there is a significant possibility of population overlap throughout our research, as many studies were conducted using the same registries with only different compounds. Fourth, we could not perform a subgroup analysis based on maternal age due to the limited data available in the included studies. Fifth, we did not analyze paternal exposure, which could be a confounding variable influencing the observed associations.

Our primary analysis findings underscore the critical need for heightened awareness and precautionary measures to protect expectant mothers from exposure to EDCs. Given the significant associations between maternal exposure to various EDCs and CHDs, particularly heavy metals, PAHs, and certain pesticides, healthcare providers should pay more attention to reducing these exposures during pregnancy. This could involve advocating for safer environmental practices, providing education on potential sources of EDCs, and encouraging lifestyle modifications that reduce contact with these harmful compounds. Moreover, regulatory authorities should consider stricter policies and monitoring systems to mitigate the release of EDCs into the environment. Public health initiatives could focus on identifying and mitigating high-risk areas, such as industrial zones and regions with heavy pesticide use, to protect susceptible populations.

Future researchers should address the limitations identified in our study, particularly the significant heterogeneity between included studies together with observed differences in the pooled evidence concerning sample and non-sampling methods. Longitudinal cohort studies with more precise exposure assessment methods, depending mainly on human sample methods with larger sample sizes, are essential to validate our findings and establish stronger causal relationships. Measuring human concentrations in humans, which integrate all sources of exposure and reflect body burden, is expected to yield a more robust relationship between maternal EDCs exposure and CHDs. Additionally, further examination into the mechanisms underlying the effects of EDCs on fetal development could provide valuable insights into potential intervention strategies. Moreover, future research should consider including detailed age-related data to enable subgroup analyses, which could provide more nuanced insights into the relationship between maternal age and the risk of congenital heart diseases. Future studies should aim to include comprehensive data on both maternal and paternal exposures to provide a more holistic understanding of the factors contributing to congenital heart diseases. By advancing our knowledge in these areas, we can develop more effective strategies to protect maternal and fetal health from the adverse effects of environmental exposure.

## 5. Conclusions

Our comprehensive meta-analysis reveals a significant association between maternal exposure to various EDCs and a heightened risk of CHDs in offspring based on the sample methods of measuring EDCs. Notably, exposure to heavy metals, PAHs, and certain pesticides showed strong associations with specific CHD subtypes. These findings highlight the urgent need for preventive measures in clinical practice to minimize EDC exposure during pregnancy. Although our study provides robust evidence, the observed heterogeneity and potential biases call for further research to strengthen these associations and improve our understanding of the underlying mechanisms. Future research should consider including detailed age-related data to enable subgroup analyses, which could provide more nuanced insights into the relationship between maternal age and the risk of congenital heart diseases. Additionally, future studies should aim to include comprehensive data on both maternal and paternal exposures to provide a more holistic understanding of the factors contributing to congenital heart diseases.

## Figures and Tables

**Table 2:** Summary of our study outcomes.

Outcome		Number of Studies	Effect Estimate Odds Ratio [95% CI]
**Any Congenital Heart Defects by Non-Sample Methods**		36	1.08 [0.93, 1.26]
	Heavy Metals	16	0.90 [0.68, 1.18]
	Phthalates	5	1.18 [0.67, 2.07]
	APC (alkylphenolic compounds)	5	1.36 [0.88, 2.12]
	BPA (bisphenol A compounds)	3	0.99 [0.52, 1.86]
	PCBs (polychlorinated biphenyls)	4	1.19 [0.74, 1.92]
	Pesticides	13	1.04 [0.64, 1.67]
	PAH (polycyclic aromatic hydrocarbons)	1	1.01 [0.89, 1.14]
	Housing renovation	3	1.56 [1.14, 2.14]
	Solvents	10	1.12 [0.90, 1.39]
	Others	7	1.01 [0.90, 1.13]
**Any Congenital Heart Defects by Sample Methods**		52	1.63 [1.35, 1.97]
	Heavy metals	38	1.91 [1.48, 2.45]
	Pesticides	2	0.51 [0.10, 2.65]
	Phthalates	8	1.02 [0.85, 1.22]
	PAH	2	2.01 [1.39, 2.91]
	Perfluoroalkyl	2	1.90 [1.34, 2.67]
**Septal Heart Defects by Non-Sample Methods**		13	1.13 [1.01, 1.26]
	Solvents	3	0.99 [0.97, 1.02]
	Pesticides	3	0.93 [0.77, 1.12]
	PAH	2	1.19 [0.93, 1.53]
	Heavy metals	2	1.41 [1.19, 1.66]
	Others	6	1.44 [1.02, 2.03]
**Septal Heart Defects by Sample Methods**		23	2.31 [1.56, 3.42]
	Heavy metals	21	2.30 [1.47, 3.59]
	Others	2	2.37 [1.50, 3.74]
**VSD by Non-Sample Methods**		25	1.17 [1.03, 1.33]
	Heavy metals	13	1.00 [0.75, 1.33]
	Pesticides	10	0.93 [0.78, 1.10]
	PCBs	4	1.45 [0.80, 2.61]
	Phthalates	4	1.63 [0.76, 3.49]
	APC	4	1.39 [0.78, 2.48]
	Solvents	4	1.21 [0.89, 1.65]
	PAH	2	1.17 [0.81, 1.69]
	Housing renovation	2	2.62 [0.83, 8.28]
	BPA	2	1.01 [0.33, 3.10]
	Others	3	1.21 [0.90, 1.64]
**ASD by Non-Sample Methods**		15	0.97 [0.78, 1.20]
	Heavy metals	8	0.75 [0.46, 1.23]
	Solvents	4	1.06 [0.85, 1.32]
	PCBs	3	1.01 [0.46, 2.23]
	BPA	3	0.89 [0.34, 2.34]
	Phthalates	3	1.20 [0.61, 2.37]
	APC	3	1.00 [0.65, 1.55]
	PAH	2	0.84 [0.49, 1.42]
	Housing renovation	2	1.27 [0.71, 2.25]
	Others	3	1.00 [0.65, 1.55]
**Conotrunca** **l** **Heart Defects by Non-sample Methods**		15	1.09 [0.96, 1.24]
	Heavy metals	5	0.89 [0.71, 1.11]
	Pesticides	3	0.98 [0.75, 1.29]
	Solvents	4	1.18 [0.88, 1.59]
	PAH	2	1.45 [1.07, 1.95]
	Others	2	1.38 [0.86, 2.20]
**Conotrunca** **l** **Heart Defects by Sample Methods**		21	2.61 [1.72, 3.96]
	Heavy metals	19	2.77 [1.74, 4.39]
	Others	2	1.74 [0.84, 3.57]
**Tetralogy of Fallot by Non-Sample Methods**		13	1.34 [1.11, 1.61]
	Pesticides	4	1.16 [0.83, 1.61]
	Heavy metals	2	1.40 [0.56, 3.53]
	PAH	2	1.74 [1.23, 2.45]
	Solvents	2	1.11 [0.73, 1.68]
	Others	5	1.77 [1.24, 2.51]
**TGA by Non-Sample Methods**		11	1.26 [1.04, 1.52]
	Pesticides	6	1.83 [1.22, 2.74]
	Heavy metals	3	0.89 [0.71, 1.11]
	Solvents	3	1.24 [0.86, 1.77]
	PCBs	2	1.42 [0.44, 4.63]
	APC	2	1.50 [0.49, 4.57]
	Phthalates	2	1.76 [0.79, 3.90]
	Others	4	0.98 [0.66, 1.47]
**Left Ventricular Outflow Tract Obstruction Defects by Non-Sample Methods**		12	1.01 [0.88, 1.15]
	Pesticides	5	1.04 [0.85, 1.26]
	Solvents	3	0.89 [0.68, 1.16]
	Phthalates	2	1.18 [0.10, 13.58]
	PAH	2	1.27 [0.91, 1.78]
	Others	4	0.91 [0.66, 1.27]
**Left Ventricular Outflow Tract Obstruction Defects by Sample Methods**		9	4.00 [2.65, 6.03]
	Heavy metals	8	4.34 [2.73, 6.90]
	PAH	1	2.66 [1.09, 6.53]
**Hypoplastic Left Heart Syndrome by Non-Sample Methods**		9	1.33 [0.83, 2.13]
	Solvents	3	0.94 [0.40, 2.22]
	Pesticides	3	2.15 [0.94, 4.90]
	PAH	2	1.17 [0.70, 1.94]
	Others	3	1.13 [0.60, 2.11]
**Coarctation of Aorta by Non-Sample Methods**		17	1.23 [0.95, 1.59]
	Heavy metals	7	1.01 [0.58, 1.74]
	Solvents	3	1.05 [0.68, 1.62]
	Pesticides	3	1.40 [0.65, 3.00]
	PAH	2	1.55 [0.93, 2.58]
	Others	5	1.49 [1.02, 2.18]
**Aortic Stenosis by Non-Sample Methods**		4	1.01 [0.56, 1.83]
**Right Ventricular Outflow Tract Obstruction Defects by Non-Sample Methods**		10	1.04 [0.88, 1.22]
	Pesticides	5	0.99 [0.65, 1.50]
	Solvents	3	1.19 [0.92, 1.54]
	PAH	2	0.61 [0.38, 0.97]
	APC	2	0.35 [0.10, 1.22]
	Phthalates	2	1.23 [0.59, 2.56]
	Others	4	1.26 [0.93, 1.70]
**Right Ventricular Outflow Tract Obstruction Defects by Sample Methods**		15	3.11 [1.65, 5.88]
	Heavy metals	14	3.17 [1.56, 6.44]
	PAH	1	2.42 [1.19, 4.93]
**Pulmonary Valve Stenosis by Non-Sample Methods**		12	1.33 [1.03, 1.73]
	Pesticides	5	1.24 [0.88, 1.75]
	Heavy metals	2	1.08 [0.31, 3.74]
	Solvents	2	1.22 [0.88, 1.68]
	Others	5	2.41 [1.21, 4.82]
**PDA by Non-Sample Methods**		9	1.68 [1.25, 2.26]
	Heavy metals	7	1.21 [0.92, 1.59]
	Pesticides	4	2.44 [1.47, 4.05]
	Phthalates	3	1.57 [0.61, 4.06]
	APC	3	1.89 [1.19, 3.00]
	BPA	2	2.49 [0.64, 9.67]
	Housing renovation	2	2.34 [1.34, 4.08]
	PCBs	2	1.85 [0.70, 4.90]
**PDA by Sample Methods**		3	2.23 [1.35, 3.67]
**TAPVR by Non-Sample Methods**		7	1.62 [0.94, 2.79]
**TAPVR by Sample Methods**		4	3.56 [1.80, 7.05]

## Data Availability

Data are available from the corresponding author upon reasonable request.

## Supplementary Materials

The following supporting information can be downloaded at: https://www.mdpi.com/article/10.3390/metabo14120709/s1. Figure S1. Forest Plot of Atrial Septal Defects (ASD) Incidence Measured by Non-Sample Methods: This forest plot shows the odds ratios and 95% confidence intervals for the association between maternal exposure to endocrine-disrupting chemicals and the incidence of atrial septal defects, based on non-sample methods of exposure assessment. Subgroups include heavy metals, pesticides, solvents, polychlorinated biphenyls, bisphenol A, phthalates, alkylphenolic compounds, polycyclic aromatic hydrocarbons, and housing renovation compounds; Figure S2. Forest Plot of Conotruncal Heart Defect Incidence Measured by Non-Sample Methods: This forest plot presents the odds ratios and 95% confidence intervals for the association between maternal exposure to endocrine-disrupting chemicals and the incidence of conotruncal heart defects, based on non-sample methods of exposure assessment. Subgroups include heavy metals, pesticides, and solvents; Figure S3. Forest Plot of Conotruncal Heart Defect Incidence Measured Using Human Samples: This forest plot displays the odds ratios and 95% confidence intervals for the association between maternal exposure to endocrine-disrupting chemicals and the incidence of conotruncal heart defects, based on human sample methods of exposure assessment. Subgroups include heavy metals and polycyclic aromatic hydrocarbons; Figure S4. Forest Plot of Tetralogy of Fallot Incidence Measured by Non-Sample Methods: This forest plot shows the odds ratios and 95% confidence intervals for the association between maternal exposure to endocrine-disrupting chemicals and the incidence of tetralogy of Fallot, based on non-sample methods of exposure assessment. Subgroups include heavy metals, pesticides, and polycyclic aromatic hydrocarbons; Figure S5. Forest Plot of Transposition of Great Arteries Incidence Measured by Non-Sample Methods: This forest plot presents the odds ratios and 95% confidence intervals for the association between maternal exposure to endocrine-disrupting chemicals and the incidence of transposition of great arteries, based on non-sample methods of exposure assessment. Subgroups include heavy metals, pesticides, solvents, polychlorinated biphenyls, alkylphenolic compounds, and phthalates; Figure S6. Forest Plot of Left Ventricular Outflow Tract Obstruction Defect Incidence Measured by Non-Sample Methods: This forest plot displays the odds ratios and 95% confidence intervals for the association between maternal exposure to endocrine-disrupting chemicals and the incidence of left ventricular outflow tract obstruction defects, based on non-sample methods of exposure assessment. Subgroups include heavy metals, pesticides, solvents, and polycyclic aromatic hydrocarbons; Figure S7. Forest Plot of Left Ventricular Outflow Tract Obstruction Defect Incidence Measured Using Human Samples: This forest plot shows the odds ratios and 95% confidence intervals for the association between maternal exposure to endocrine-disrupting chemicals and the incidence of left ventricular outflow tract obstruction defects, based on human sample methods of exposure assessment. Subgroups include heavy metals and polycyclic aromatic hydrocarbons; Figure S8. Forest Plot of Hypoplastic Left Heart Syndrome Incidence Measured by Non-Sample Methods: This forest plot presents the odds ratios and 95% confidence intervals for the association between maternal exposure to endocrine-disrupting chemicals and the incidence of hypoplastic left heart syndrome, based on non-sample methods of exposure assessment. Subgroups include heavy metals, pesticides, and polycyclic aromatic hydrocarbons; Figure S9. Forest Plot of Coarctation of Aorta Incidence Measured by Non-Sample Methods: This forest plot displays the odds ratios and 95% confidence intervals for the association between maternal exposure to endocrine-disrupting chemicals and the incidence of coarctation of the aorta, based on non-sample methods of exposure assessment. Subgroups include heavy metals, pesticides, solvents, and polycyclic aromatic hydrocarbons; Figure S10. Forest Plot of Aortic Stenosis Incidence Measured by Non-Sample Methods: This forest plot shows the odds ratios and 95% confidence intervals for the association between maternal exposure to endocrine-disrupting chemicals and the incidence of aortic stenosis, based on non-sample methods of exposure assessment. Subgroups include heavy metals, pesticides, and solvents; Figure S11. Forest Plot of Right Ventricular Outflow Tract Obstruction Defect Incidence Measured by Non-Sample Methods: This forest plot presents the odds ratios and 95% confidence intervals for the association between maternal exposure to endocrine-disrupting chemicals and the incidence of right ventricular outflow tract obstruction defects, based on non-sample methods of exposure assessment. Subgroups include heavy metals, pesticides, solvents, alkylphenolic compounds, and phthalates; Figure S12. Forest Plot of Right Ventricular Outflow Tract Obstruction Defect Incidence Measured Using Human Samples: This forest plot displays the odds ratios and 95% confidence intervals for the association between maternal exposure to endocrine-disrupting chemicals and the incidence of right ventricular outflow tract obstruction defects, based on human sample methods of exposure assessment. Subgroups include heavy metals and polycyclic aromatic hydrocarbons; Figure S13. Forest Plot of Pulmonary Valve Stenosis Incidence Measured by Non-Sample Methods: This forest plot shows the odds ratios and 95% confidence intervals for the association between maternal exposure to endocrine-disrupting chemicals and the incidence of pulmonary valve stenosis, based on non-sample methods of exposure assessment. Subgroups include heavy metals, pesticides, and solvents; Figure S14. Forest Plot of Patent Ductus Arteriosus (PDA) Incidence Measured by Non-Sample Methods: This forest plot presents the odds ratios and 95% confidence intervals for the association between maternal exposure to endocrine-disrupting chemicals and the incidence of patent ductus arteriosus, based on non-sample methods of exposure assessment. Subgroups include heavy metals, pesticides, alkylphenolic compounds, bisphenol A, and polychlorinated biphenyls; Figure S15. Forest Plot of Patent Ductus Arteriosus (PDA) Incidence Measured Using Human Samples: This forest plot displays the odds ratios and 95% confidence intervals for the association between maternal exposure to endocrine-disrupting chemicals and the incidence of patent ductus arteriosus, based on human sample methods of exposure assessment. Subgroups include heavy metals; Figure S16. Forest Plot of Total Anomaly Pulmonary Venous Return (TAPVR) Incidence Measured by Non-Sample Methods: This forest plot shows the odds ratios and 95% confidence intervals for the association between maternal exposure to endocrine-disrupting chemicals and the incidence of total anomaly pulmonary venous return, based on non-sample methods of exposure assessment. Subgroups include heavy metals, pesticides, and solvents; Figure S17. Forest Plot of Total Anomaly Pulmonary Venous Return (TAPVR) Incidence Measured Using Human Samples: This forest plot presents the odds ratios and 95% confidence intervals for the association between maternal exposure to endocrine-disrupting chemicals and the incidence of total anomaly pulmonary venous return, based on human sample methods of exposure assessment. Subgroups include heavy metals; Table S1: PRISMA checklist; Table S2: Search strategy in the different databases; Table S3: Quality assessment of the included case–control studies; Table S4: Quality assessment of the included cohort studies.

## Abbreviations

ASD	Atrial Septal Defect
APC	Alkyl Phenolic Compounds
BPA	Bisphenol A
BPC	Biphenolic Compounds
CHD	Congenital Heart Disease
CI	Confidence Interval
EDC	Endocrine-Disrupting Chemicals
FFQ	Food Frequency Questionnaire
ICD	International Classification of Diseases
LVOTOD	Left Ventricular Outflow Tract Obstruction Defect
NOS	Newcastle–Ottawa Scale
OR	Odds Ratio
PAH	Polycyclic Aromatic Hydrocarbons
PCB	Polychlorinated Biphenyls
PDA	Patent Ductus Arteriosus
PRISMA	Preferred Reporting Items for Systematic Reviews and Meta-Analyses
RVOTOD	Right Ventricular Outflow Tract Obstruction Defect
SES	Socioeconomic Status
TAPVR	Total Anomalous Pulmonary Venous Return
TGA	Transposition of Great Arteries
TOF	Tetralogy of Fallot
VSD	Ventricular Septal Defect
