# Supplementary material for: The Relationship Between Maternal Exposure to Endocrine-Disrupting Chemicals and the Incidence of Congenital Heart Diseases: A Systematic Review and Meta-Analysis
Source: Metabolites. 2024 Dec 16;14(12):709. doi: 10.3390/metabo14120709 (PMC11676353; doi:10.3390/metabo14120709)
Supplement: Supplementary file 1 [file metabolites-14-00709-s001.zip › Supplementary Table S2.pdf]

Supplementary Table S2: Search strategy in the different databases

Databases	Search Strategy	Results
**Pubmed**	("Endocrine disrupting chemical" OR "Endocrine disrupting chemicals" OR "occupational exposure" OR dichlorodiphenyldichloroethylene OR Hexachlorobenzene OR "Polychlorinated compounds" OR "Polychlorinated compound" OR Solvent OR Solvents OR "polycyclic aromatic hydrocarbons" OR "polycyclic aromatic hydrocarbon" OR "polychlorinated biphenyls" OR "polychlorinated biphenyl" OR Herbicides OR Herbicide OR Rodenticides OR Rodenticide OR Neonicotinoids OR Neonicotinoid OR Atrazine OR Pesticide OR Pesticides OR Phthalates OR Phthalate OR Alkylphenol OR Bisphenol OR perfluoroalkyl OR Triclosan OR "Heavy metals" OR "Heavy metal" OR "Lead Exposure" OR Arsenic OR Cadmium OR Mercury OR Chromium OR Copper OR Zinc OR aluminum OR Nickel OR Cobalt OR Titanium) AND ("congenital heart disease" OR "congenital heart diseases" OR "congenital heart defects" OR "congenital heart defect" OR "Tetralogy of Fallot" OR "Transposition of the great arteries" OR "Anomalous pulmonary venous return" OR "Hypoplastic left heart syndrome" OR "Coarctation of the aorta" OR "Aortic stenosis" OR "Pulmonary stenosis" OR "Ventricular septal defect" OR "Atrial septal defect" OR "Conotruncal defects" OR "Conotruncal defect" OR "Pulmonary Atresia" OR "Tricuspid atresia" OR "Bicuspid aortic valve" OR "Truncus arteriosus" OR "Ductus arteriosus" OR "Ebstein anomaly")	1022
**Scopus**	TITLE-ABS-KEY (("endocrine disrupting chemical" OR "endocrine disrupting chemicals" OR "occupational exposure" OR dichlorodiphenyldichloroethylene OR hexachlorobenzene OR "polychlorinated compounds" OR "polychlorinated compound" OR solvent OR solvents OR "polycyclic aromatic hydrocarbons" OR "polycyclic aromatic hydrocarbon" OR "polychlorinated biphenyls" OR "polychlorinated biphenyl" OR herbicides OR herbicide OR rodenticides OR rodenticide OR neonicotinoids OR neonicotinoid OR atrazine OR pesticide OR pesticides OR phthalates OR phthalate OR alkylphenol OR bisphenol OR perfluoroalkyl OR triclosan OR "heavy metals" OR "heavy metal" OR "lead exposure" OR arsenic OR cadmium OR mercury OR chromium OR copper OR zinc OR aluminum OR nickel OR cobalt OR titanium) AND ("congenital heart disease" OR "congenital heart diseases" OR "congenital heart defects" OR "congenital heart defect" OR "tetralogy of fallot" OR "transposition of the great arteries" OR "anomalous pulmonary venous return" OR "hypoplastic left heart syndrome" OR "coarctation of the aorta" OR "aortic stenosis" OR "pulmonary stenosis" OR "ventricular septal defect" OR "atrial septal defect" OR "conotruncal defects" OR "conotruncal defect" OR "pulmonary atresia" OR "tricuspid atresia" OR "bicuspid aortic valve" OR "truncus arteriosus" OR "ductus arteriosus" OR "ebstein anomaly"))	1039

**Web of science**	(ALL=(("Endocrine disrupting chemical" OR "Endocrine disrupting chemicals" OR "occupational exposure" OR dichlorodiphenyldichloroethylene OR Hexachlorobenzene OR "Polychlorinated compounds" OR "Polychlorinated compound" OR Solvent OR Solvents OR "polycyclic aromatic hydrocarbons" OR "polycyclic aromatic hydrocarbon" OR "polychlorinated biphenyls" OR "polychlorinated biphenyl" OR Herbicides OR Herbicide OR Rodenticides OR Rodenticide OR Neonicotinoids OR Neonicotinoid OR Atrazine OR Pesticide OR Pesticides OR Phthalates OR Phthalate OR Alkylphenol OR Bisphenol OR perfluoroalkyl OR Triclosan OR "Heavy metals" OR "Heavy metal" OR "Lead Exposure" OR Arsenic OR Cadmium OR Mercury OR Chromium OR Copper OR Zinc OR aluminum OR Nickel OR Cobalt OR Titanium))) AND ALL=(("congenital heart disease" OR "congenital heart diseases" OR "congenital heart defects" OR "congenital heart defect" OR "Tetralogy of Fallot" OR "Transposition of the great arteries" OR "Anomalous pulmonary venous return" OR "Hypoplastic left heart syndrome" OR "Coarctation of the aorta" OR "Aortic stenosis" OR "Pulmonary stenosis" OR "Ventricular septal defect" OR "Atrial septal defect" OR "Conotruncal defects" OR "Conotruncal defect" OR "Pulmonary Atresia" OR "Tricuspid atresia" OR "Bicuspid aortic valve" OR "Truncus arteriosus" OR "Ductus arteriosus" OR "Ebstein anomaly"))	605
**Cochrane library**	("Endocrine disrupting chemical" OR "Endocrine disrupting chemicals" OR "occupational exposure" OR dichlorodiphenyldichloroethylene OR Hexachlorobenzene OR "Polychlorinated compounds" OR "Polychlorinated compound" OR Solvent OR Solvents OR "polycyclic aromatic hydrocarbons" OR "polycyclic aromatic hydrocarbon" OR "polychlorinated biphenyls" OR "polychlorinated biphenyl" OR Herbicides OR Herbicide OR Rodenticides OR Rodenticide OR Neonicotinoids OR Neonicotinoid OR Atrazine OR Pesticide OR Pesticides OR Phthalates OR Phthalate OR Alkylphenol OR Bisphenol OR perfluoroalkyl OR Triclosan OR "Heavy metals" OR "Heavy metal" OR "Lead Exposure" OR Arsenic OR Cadmium OR Mercury OR Chromium OR Copper OR Zinc OR aluminum OR Nickel OR Cobalt OR Titanium) AND ("congenital heart disease" OR "congenital heart diseases" OR "congenital heart defects" OR "congenital heart defect" OR "Tetralogy of Fallot" OR "Transposition of the great arteries" OR "Anomalous pulmonary venous return" OR "Hypoplastic left heart syndrome" OR "Coarctation of the aorta" OR "Aortic stenosis" OR "Pulmonary stenosis" OR "Ventricular septal defect" OR "Atrial septal defect" OR "Conotruncal defects" OR "Conotruncal defect" OR "Pulmonary Atresia" OR "Tricuspid atresia" OR "Bicuspid aortic valve" OR "Truncus arteriosus" OR "Ductus arteriosus" OR "Ebstein anomaly") in Title Abstract Keyword - (Word variations have been searched)	38
**The total from the four databases:**		2704
**Number of duplicates:**		669
**Number after removing duplication: (By Endnote):**		2035
