# Supplementary material for: The Relationship Between Maternal Exposure to Endocrine-Disrupting Chemicals and the Incidence of Congenital Heart Diseases: A Systematic Review and Meta-Analysis
Source: Metabolites. 2024 Dec 16;14(12):709. doi: 10.3390/metabo14120709 (PMC11676353; doi:10.3390/metabo14120709)
Supplement: Supplementary file 1 [file metabolites-14-00709-s001.zip › Supplementary Table S3.pdf]

Study ID	Supplementary Table S3: Quality assessment of the included Case Control Studies								
	Selection				Comparability	Exposure			Quality Score
	Is the case definition adequate?	Representativeness of the cases	Selection of Controls	Definition of Controls	Comparability of cases and controls on the basis of the design or analysis	Ascertainment of exposure	Same method of ascertainment for cases and controls	Non-Response rate	
**Batra 2007**	*	*	*	*	*	*	*	*	Good
**Brender 2014**	*	*	*	*	*	*	*	*	Good
**Carmichael 2014**	*	*		*	*	*	*	*	Good
**Cresci 2011**	*	*	*	*	*	*	*	*	Good
**Fazekas-Pongor 2020**	*	*	*	*	*	*	*	*	Good
**Fazekas-Pongor 2021**	*	*	*	*	*	*	*	*	Good
**Fixler 1998**	*	*	*	*	*	*	*	*	Good
**Garcia 1998**	*		*	*		*	*	*	Moderate
**Gilboa 2012**	*	*	*	*	*	*	*	*	Good
**Hu 2014**	*	*	*	*	*	*	*	*	Good
**Huang 2023**	*	*	*	*	* *	*	*	*	Good
**Jin 2016**	*	*	*	*	*	*	*	*	Good
**Kim 2017**	*	*		*	*	*	*	*	Good
**Li 2018**	*	*	*	*	*	*	*	*	Good
**Li 2024**	*	*	*	*	*	*	*	*	Good
**Liu 2013**	*	*	*	*	*	*	*	*	Good
**Liu 2015 a**	*	*	*	*	*	*	*	*	Good
**Liu 2015 b**	*	*	*			*	*	*	Moderate
**Loffredo 2001**	*	*	*	*	*	*	*	*	Good
**Luan 2023**	*	*	*	*	* *	*	*	*	Good
**Lupo 2012**	*	*	*	*	*	*	*	*	Good
**Nana Li 2024**	*	*	*	*	*	*	*	*	Good
**Nie 2020**	*	*	*	*	* *	*	*	*	Good
**Nie 2024**	*	*	*	*	*	*	*	*	Good
**Ou 2017**	*	*	*	*	* *	*	*	*	Good
**Ou 2021**	*	*	*	*	*	*	*	*	Good
**Patel 2020**	*	*	*	*	*	*	*	*	Good
**Qu 2022**	*	*	*	*	*	*	*	*	Good
**Qu 2024**	*	*	*	*	* *	*	*	*	Good
**Rappazzo 2018**	*	*	*			*	*	*	Moderate
**Rocheleau 2015**	*	*	*	*	*	*	*	*	Good
**Rudnai 2014**	*	*	*	*		*	*	*	Good
**Sainan Li 2024**	*	*	*	*	*	*	*	*	Good
**Salehi 2022**	*	*	*	*	*	*	*	*	Good
**Shaw 1999**	*	*	*	*	*	*	*	*	Good
**Snijder 2012**	*	*	*	*	*	*	*	*	Good
**Spinder 2020**	*	*		*	*	*	*	*	Good

**Suhl 2022**	*	*	*	*	*	*	*	*	Good
**Sun 2022**	*	*	*	*	* *	*	*	*	Good
**Tao 2019**	*	*	*	*	*	*	*	*	Good
**Tikkanen 1988**	*	*				*	*	*	Moderate
**Tikkanen 1991**	*	*	*	*	*	*	*	*	Good
**Tikkanen 1992**	*	*	*	*	*	*	*	*	Good
**Tikkanen 1993**	*	*		*		*	*	*	Moderate
**Villasenior 1991**	*	*		*		*	*	*	Moderate
**Wang 2013**	*	*	*	*	*	*	*	*	Good
**Wang 2015**	*	*	*	*	* *	*	*	*	Good
**Wang 2022**	*	*		*		*	*	*	Moderate
**Xiao 2023**	*	*		*	*	*	*	*	Good
**Yang 2022**	*	*	*	*	*	*	*	*	Good
**Zhang 2017**	*	*	*	*		*	*	*	Moderate
**Zhang 2019**	*	*	*	*		*	*	*	Moderate
**Zhang 2020**	*	*	*	*		*	*	*	Moderate
**Zierler 1988**	*	*		*		*	*	*	Moderate
