# Supplementary material for: The Relationship Between Maternal Exposure to Endocrine-Disrupting Chemicals and the Incidence of Congenital Heart Diseases: A Systematic Review and Meta-Analysis
Source: Metabolites. 2024 Dec 16;14(12):709. doi: 10.3390/metabo14120709 (PMC11676353; doi:10.3390/metabo14120709)
Supplement: Supplementary file 1 [file metabolites-14-00709-s001.zip › Supplementary Table S4.pdf]

Study ID	Supplementary Table S4: Quality assessment of the included Cohort studies								
	**Selection**				**Comparability**	**Outcome**			**Quality Score**
	**Representativeness of the exposed cohort**	**Selection of the non-exposed cohort**	**Ascertainment of exposure**	**Demonstration that outcome of interest was not present at start of study**	**Comparability of cohorts on the basis of the design or analysis**	**Assessment of outcome**	**Was follow-up long enough for outcomes to occur**	**Adequacy of follow up of cohorts**	
**Forand 2011**	*	*	*			*	*	*	Moderate
**Gong 2017**	*	*	*		*	*	*	*	Good
**Motoki 2019**	*	*	*		*	*	*	*	Good
**Richter 2022**	*	*	*		*	*	*	*	Good
**Zhang 2020**	*	*	*			*	*	*	Moderate
