# Supplementary figures and images for: The Relationship Between Maternal Exposure to Endocrine-Disrupting Chemicals and the Incidence of Congenital Heart Diseases: A Systematic Review and Meta-Analysis
Source: Metabolites. 2024 Dec 16;14(12):709. doi: 10.3390/metabo14120709 (PMC11676353; doi:10.3390/metabo14120709)

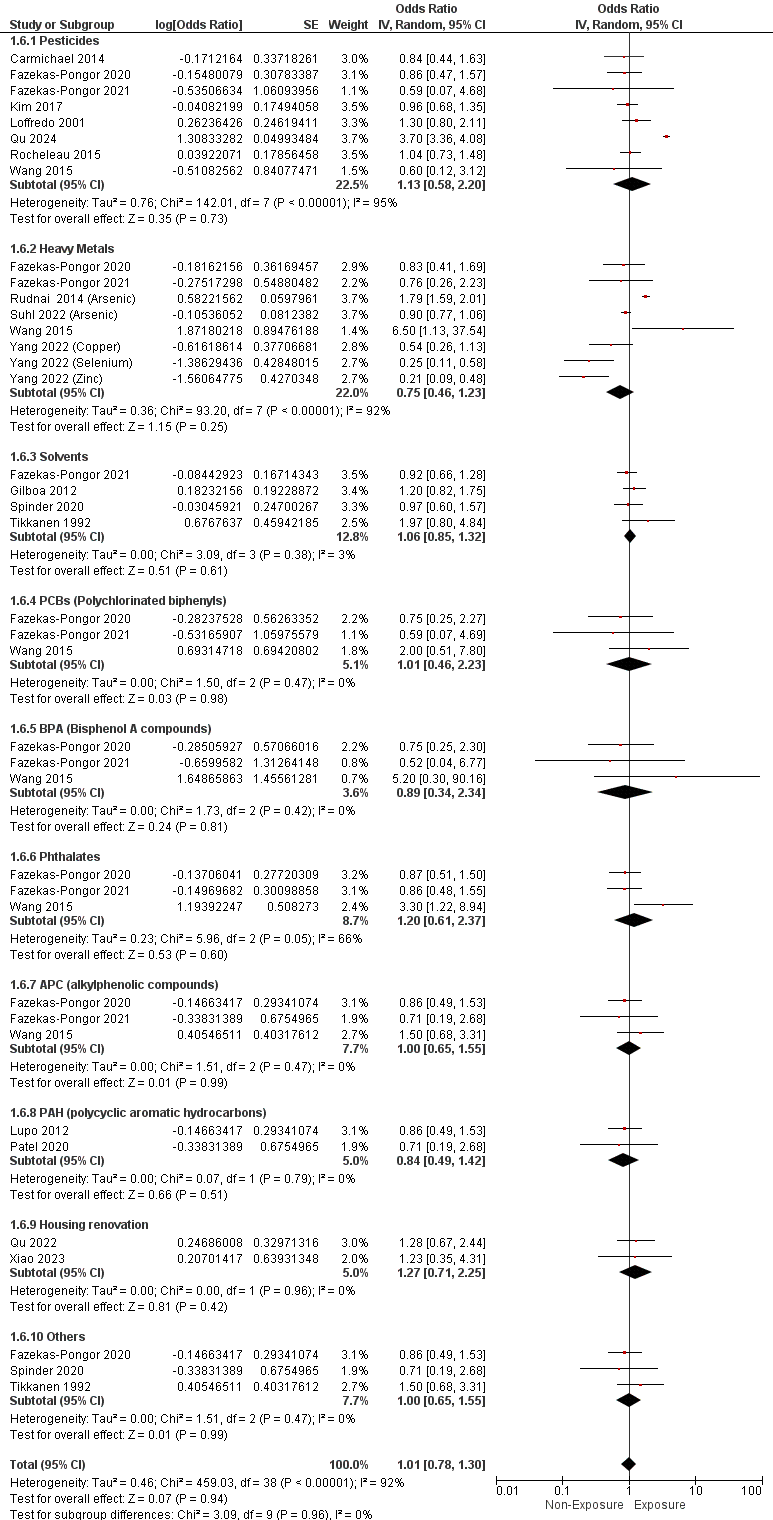

Supplement: Supplementary file 1 [file metabolites-14-00709-s001.zip › Supplementary Figure S1.png]

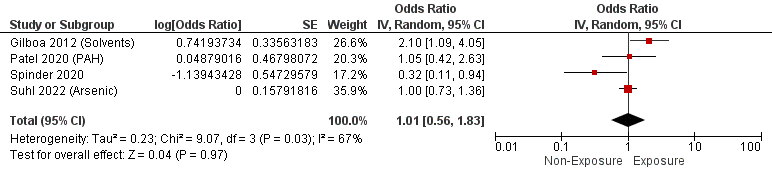

Supplement: Supplementary file 1 [file metabolites-14-00709-s001.zip › Supplementary Figure S10.png]

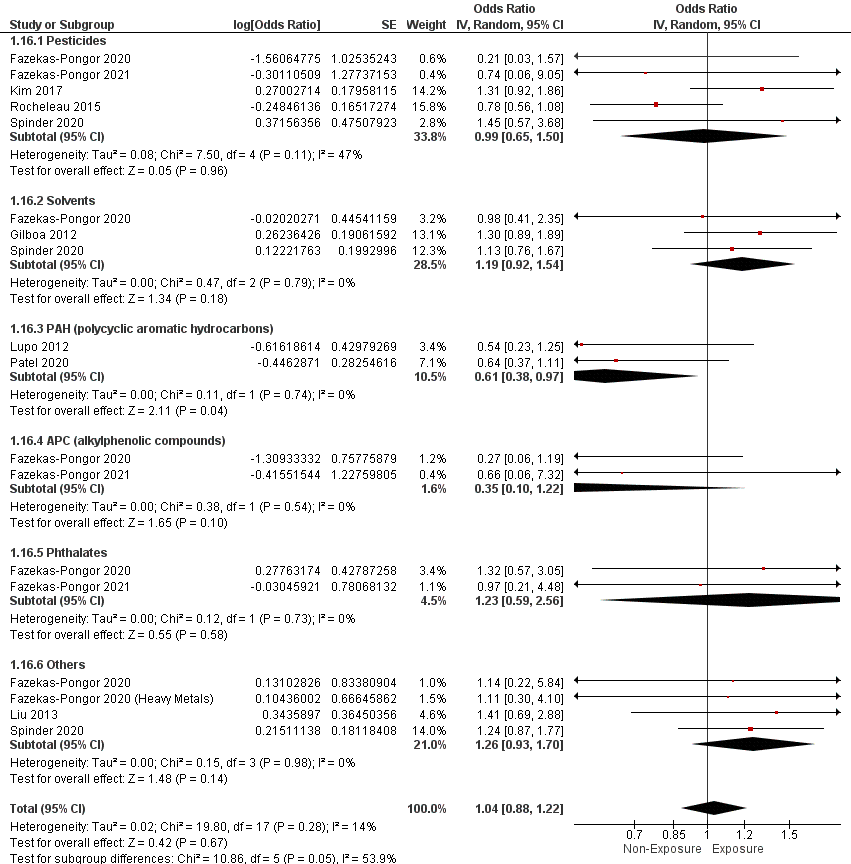

Supplement: Supplementary file 1 [file metabolites-14-00709-s001.zip › Supplementary Figure S11.png]

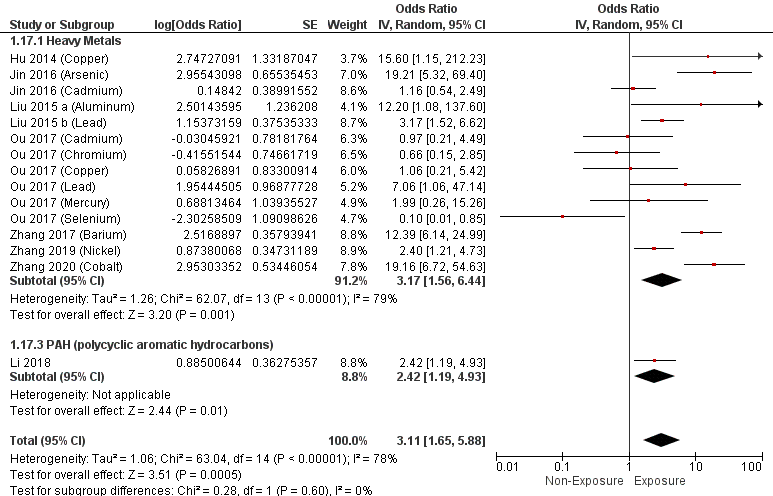

Supplement: Supplementary file 1 [file metabolites-14-00709-s001.zip › Supplementary Figure S12.png]

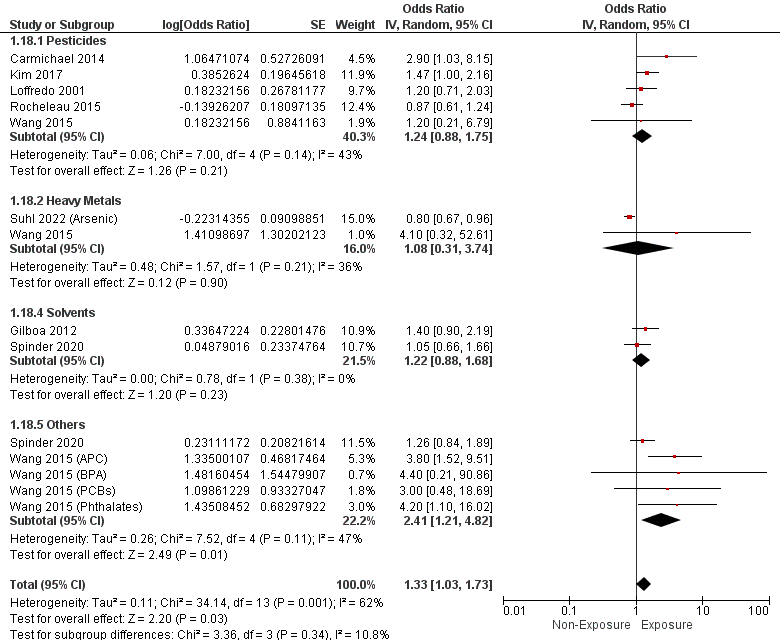

Supplement: Supplementary file 1 [file metabolites-14-00709-s001.zip › Supplementary Figure S13.png]

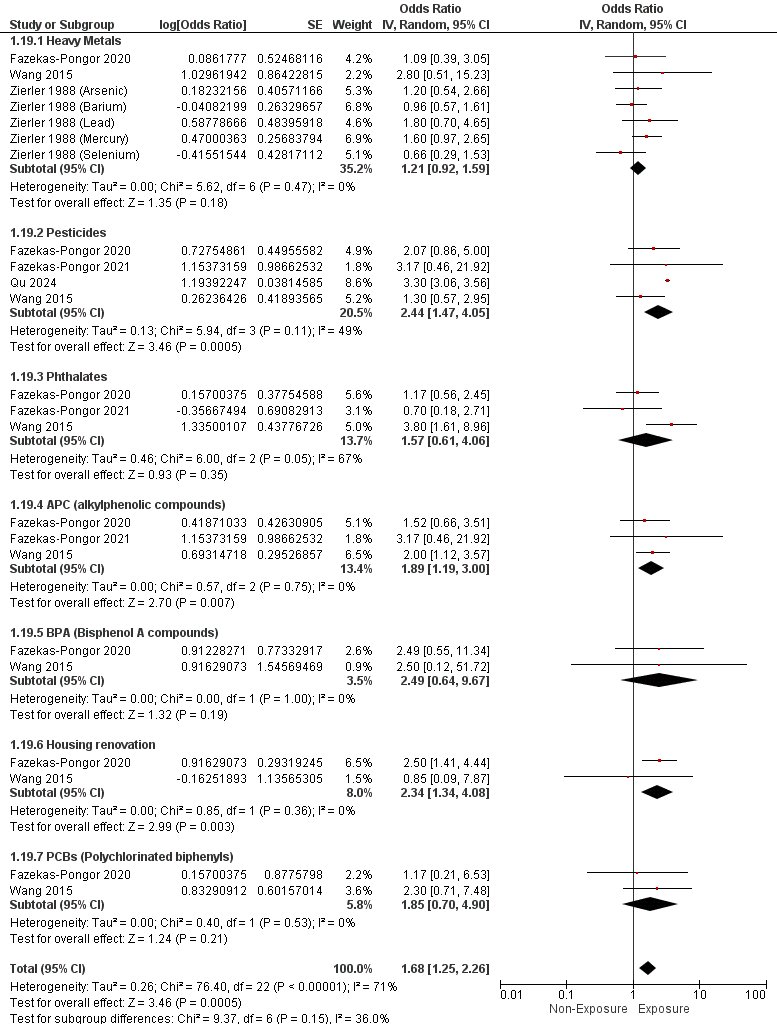

Supplement: Supplementary file 1 [file metabolites-14-00709-s001.zip › Supplementary Figure S14.png]

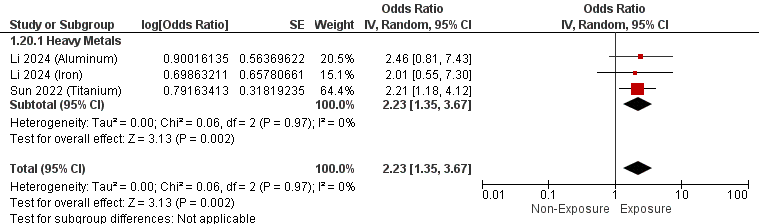

Supplement: Supplementary file 1 [file metabolites-14-00709-s001.zip › Supplementary Figure S15.png]

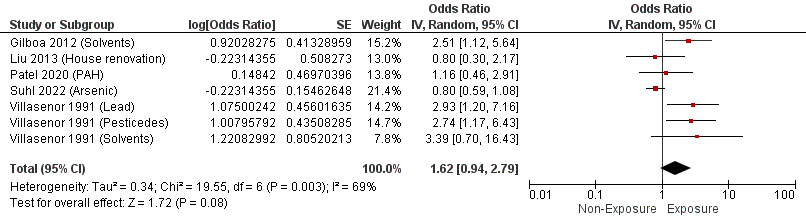

Supplement: Supplementary file 1 [file metabolites-14-00709-s001.zip › Supplementary Figure S16.png]

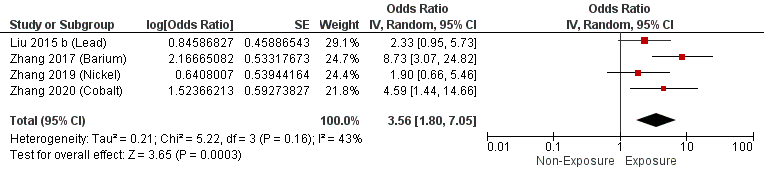

Supplement: Supplementary file 1 [file metabolites-14-00709-s001.zip › Supplementary Figure S17.png]

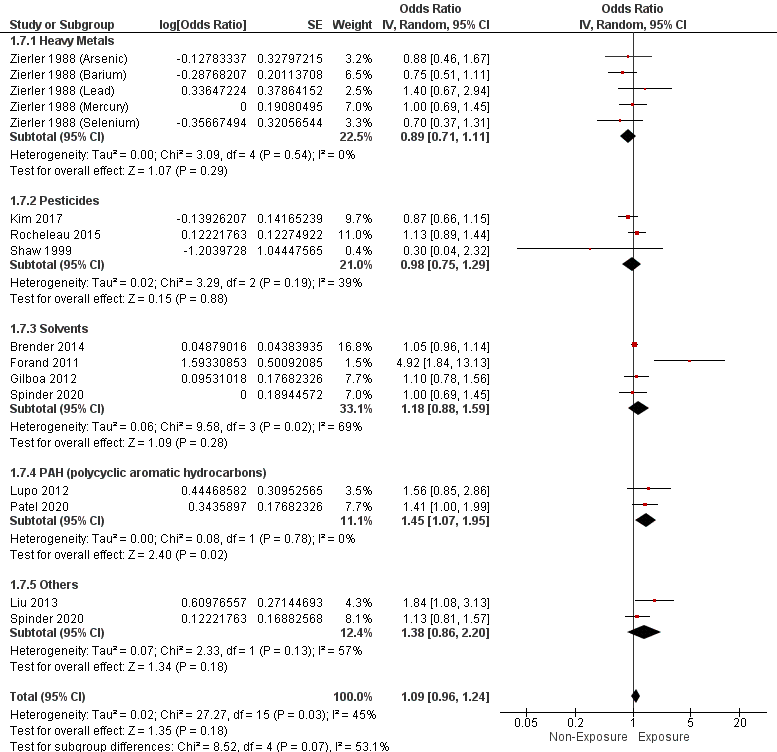

Supplement: Supplementary file 1 [file metabolites-14-00709-s001.zip › Supplementary Figure S2.png]

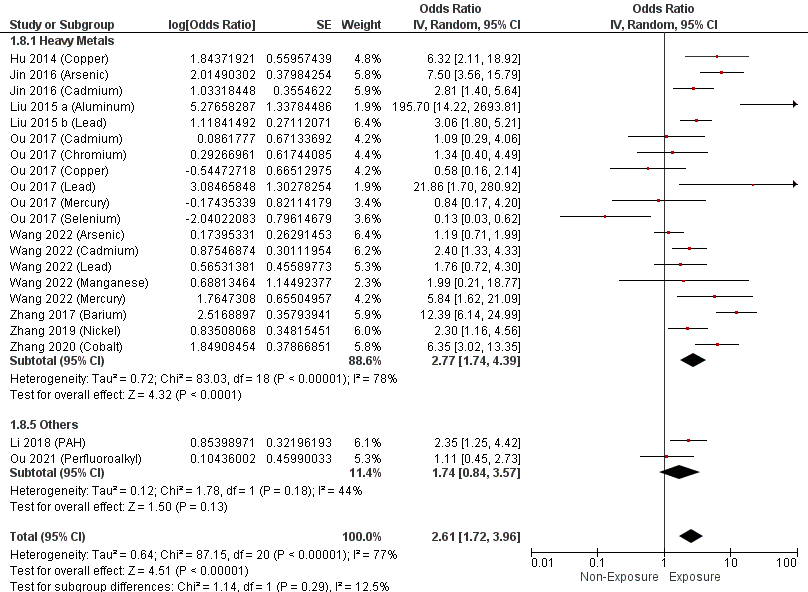

Supplement: Supplementary file 1 [file metabolites-14-00709-s001.zip › Supplementary Figure S3.png]

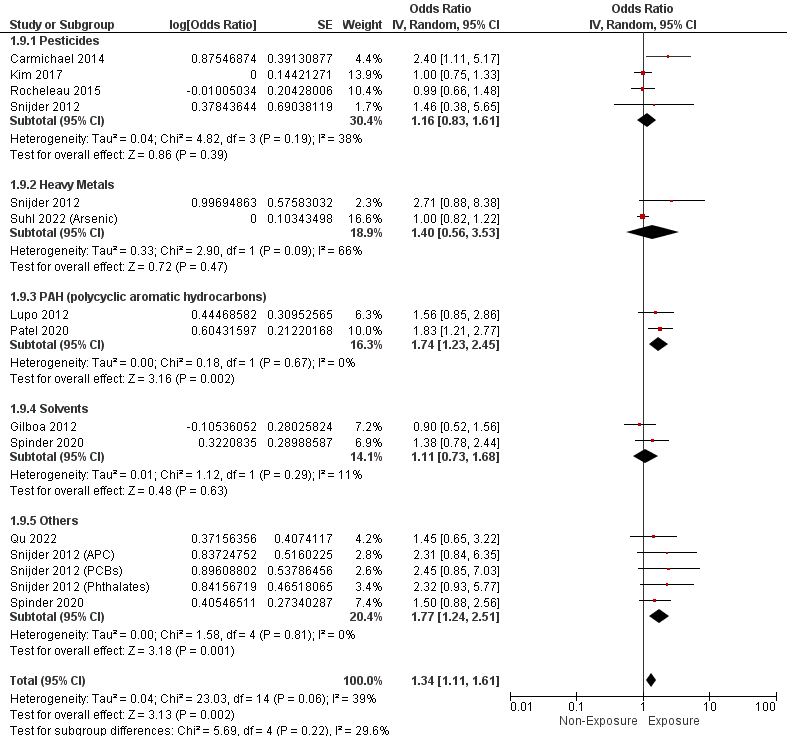

Supplement: Supplementary file 1 [file metabolites-14-00709-s001.zip › Supplementary Figure S4.png]

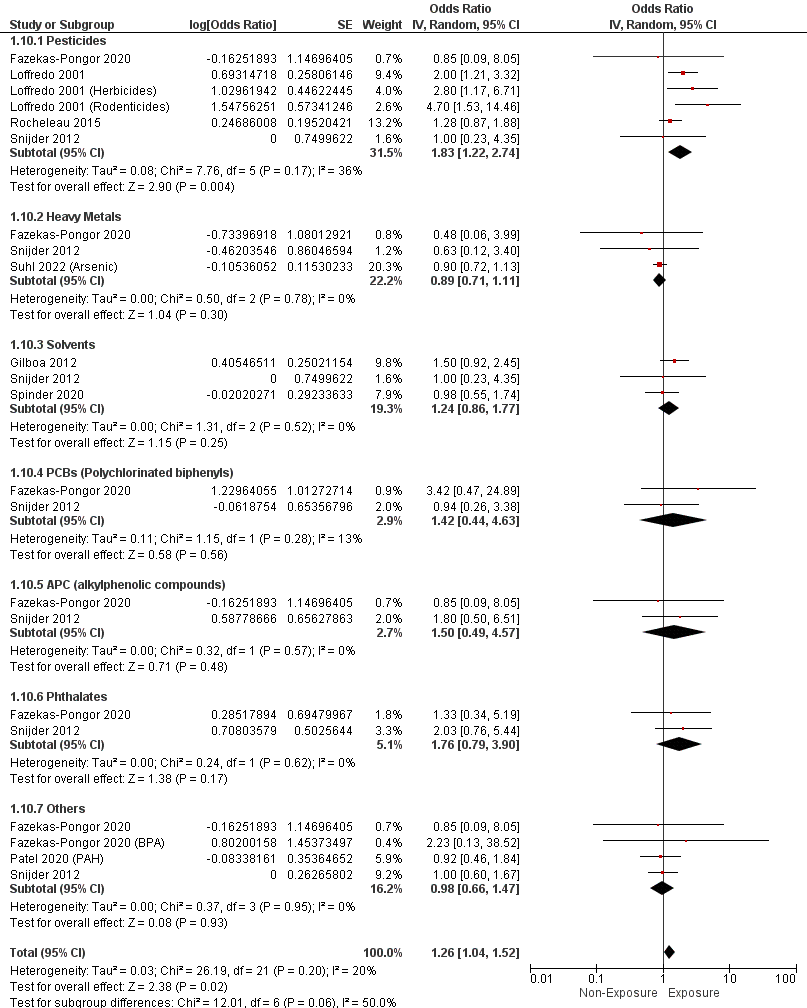

Supplement: Supplementary file 1 [file metabolites-14-00709-s001.zip › Supplementary Figure S5.png]

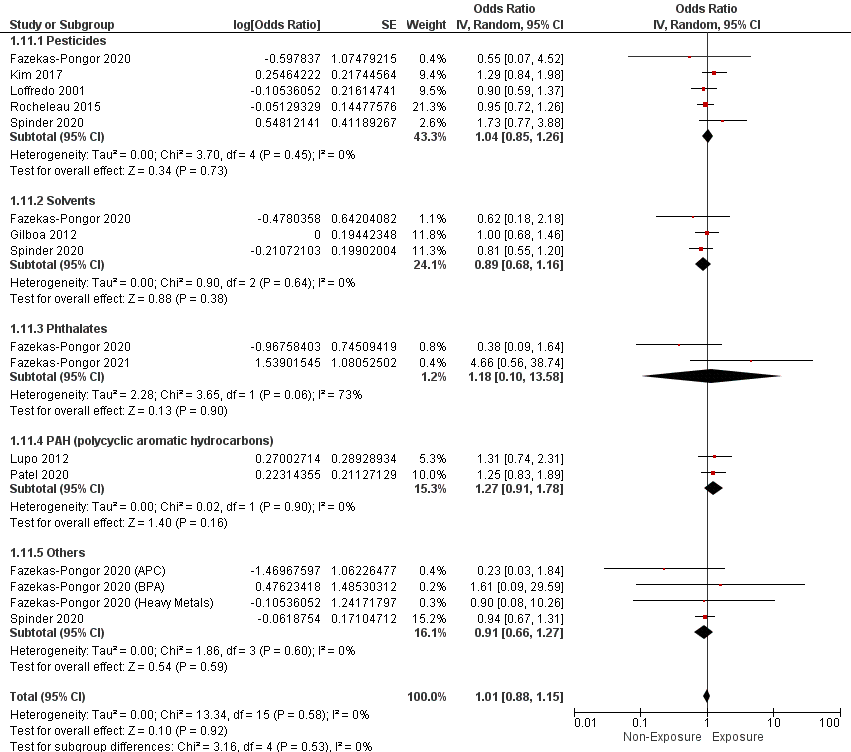

Supplement: Supplementary file 1 [file metabolites-14-00709-s001.zip › Supplementary Figure S6.png]

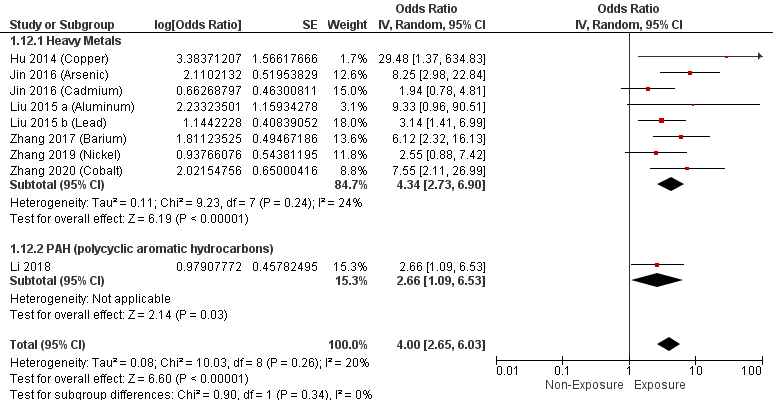

Supplement: Supplementary file 1 [file metabolites-14-00709-s001.zip › Supplementary Figure S7.png]

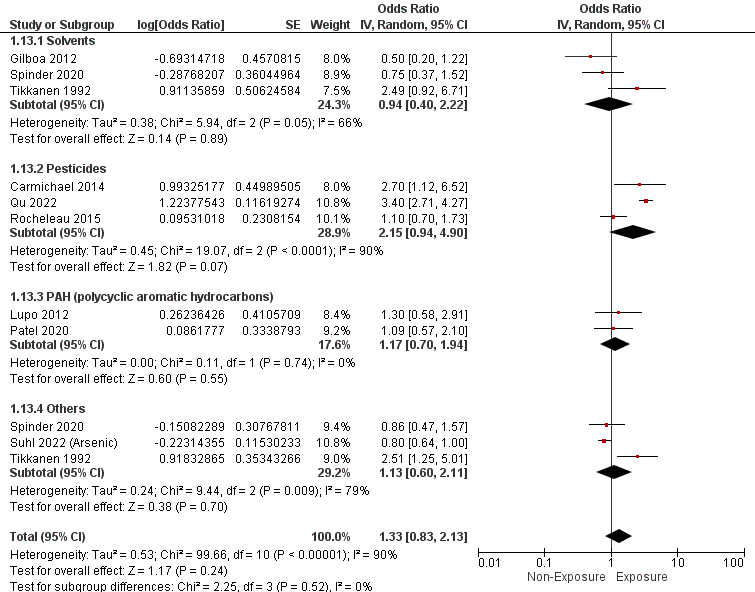

Supplement: Supplementary file 1 [file metabolites-14-00709-s001.zip › Supplementary Figure S8.png]

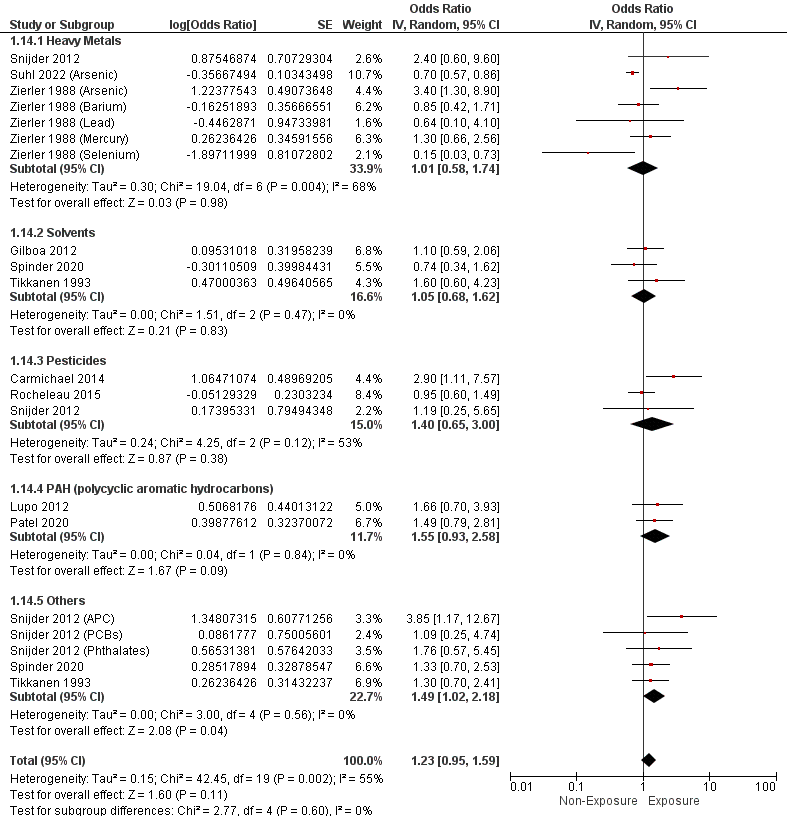

Supplement: Supplementary file 1 [file metabolites-14-00709-s001.zip › Supplementary Figure S9.png]
